# Synthesis of Very-Long-Chain Fatty Acids in the Epidermis Controls Plant Organ Growth by Restricting Cell Proliferation

**DOI:** 10.1371/journal.pbio.1001531

**Published:** 2013-04-09

**Authors:** Takashi Nobusawa, Yoko Okushima, Noriko Nagata, Mikiko Kojima, Hitoshi Sakakibara, Masaaki Umeda

**Affiliations:** 1 Graduate School of Biological Sciences, Nara Institute of Science and Technology, Ikoma, Nara, Japan; 2 Faculty of Science, Japan Women's University, Bunkyo-ku, Tokyo, Japan; 3 RIKEN Plant Science Center, Tsurumi, Yokohama, Japan; 4 JST, CREST, Ikoma, Nara, Japan; The Salk Institute for Biological Studies, United States of America

## Abstract

Plant organ growth is controlled by inter-cell-layer communication, which thus determines the overall size of the organism. The epidermal layer interfaces with the environment and participates in both driving and restricting growth via inter-cell-layer communication. However, it remains unknown whether the epidermis can send signals to internal tissue to limit cell proliferation in determinate growth. Very-long-chain fatty acids (VLCFAs) are synthesized in the epidermis and used in the formation of cuticular wax. Here we found that VLCFA synthesis in the epidermis is essential for proper development of *Arabidopsis thaliana*. Wild-type plants treated with a VLCFA synthesis inhibitor and *pasticcino* mutants with defects in VLCFA synthesis exhibited overproliferation of cells in the vasculature or in the rib zone of shoot apices. The decrease of VLCFA content increased the expression of *IPT3*, a key determinant of cytokinin biosynthesis in the vasculature, and, indeed, elevated cytokinin levels. These phenotypes were suppressed in *ipt3;5;7* triple mutants, and also by vasculature-specific expression of cytokinin oxidase, which degrades active forms of cytokinin. Our results imply that VLCFA synthesis in the epidermis is required to suppress cytokinin biosynthesis in the vasculature, thus fine-tuning cell division activity in internal tissue, and therefore that shoot growth is controlled by the interaction between the surface (epidermis) and the axis (vasculature) of the plant body.

## Abstract 2

The synthesis of very-long-chain fatty acids (VLCFAs) in the epidermis is essential for the proper control of cell growth in *Arabidopsis*. VLCFAs act via their ability to suppress cytokinin synthesis in the vasculature, thus preventing cell overproliferation in internal tissues.

## Author Summary

The epidermis functions as an important interface with the environment, but in plants it is also essential for establishing and maintaining the primary plant body. Recent studies have shown that the epidermis participates in both driving and restricting plant growth via inter-cell-layer communication. However, it remains an open question as to whether the epidermis can send signals to internal plant tissues to control cell proliferation during development. Here we report that the synthesis of very-long-chain fatty acids (VLCFAs) in the epidermis is essential for the proper control of cell proliferation in the plant *Arabidopsis thaliana*. We find that defects in VLCFA synthesis cause cells in the vasculature or in the rib zone of shoot apices to overproliferate. When VLCFA levels decrease, we observe that the synthesis of the phytohormone cytokinin increases in the vasculature. We also find that when cytokinin is degraded by the expression of cytokinin oxidase in the vasculature, enhanced cell proliferation in internal tissues is suppressed, indicating that VLCFA synthesis in the epidermis is required to suppress cytokinin biosynthesis and thus cell overproliferation. Our results demonstrate that shoot growth is controlled by interactions between the surface (epidermis) and the axis (vasculature) of the plant body, and highlight a role for VLCFAs in this interaction.

## Introduction

The epidermis is formed from the outermost L1 layer in the shoot apical meristem (SAM) and functions as an important interface with the environment. However, recent studies have shown that it also plays an essential role in the establishment and maintenance of the plant body. *Arabidopsis* mutants with defects in epidermal cell specification exhibit disorganized morphology [1]–[3]. Biophysical manipulation of the epidermis revealed that it generates mechanical constraints on inner layers, thus restricting plant growth [4],[5]. Another report showed that epidermis-specific expression of brassinosteroid receptor (BR) or brassinosteroid biosynthesis enzyme rescued plant growth in dwarf mutants, indicating that a BR-generated signal from the epidermis promotes the growth of ground tissue [6]. These results suggest that the epidermis participates in both driving and restricting growth via inter-cell-layer communication. However, it remains an open question as to whether the L1 layer can send signals to internal tissue to control cell proliferation during development.

A characteristic feature of the epidermis is that it is covered with a hydrophobic barrier, the cuticle, which prevents plants from transpiring and protects tissues from pathogen attack [7]. The cuticle is mainly composed of cutin matrix and cuticular wax; cutin is a plant-specific lipid polymer that consists of long-chain fatty acids (LCFAs) with an acyl chain length of 16 or 18 carbons, whereas cuticular wax contains very-long-chain fatty acids (VLCFAs) with fully saturated unbranched hydrocarbon chains (≥20 carbons). Plant VLCFAs are synthesized in the endoplasmic reticulum by sequential addition of 2-carbon moieties to the 18-carbon LCFA, which is made in the plastid. The carbon donor malonyl-CoA is synthesized from acetyl-CoA by acetyl-CoA carboxylase and used for each cycle of the elongation reaction. VLCFA synthesis consists of four enzymatic steps: (1) condensation of acyl-CoA with malonyl-CoA catalyzed by ketoacyl-CoA synthase (KCS), (2) reduction of 3-ketoacyl-CoA by 3-ketoacyl-CoA reductase (KCR), (3) dehydration of 3-hydroxyacyl-CoA by 3-hydroxy acyl-CoA dehydratase (HCD), and (4) reduction of enoyl-CoA by enoyl-CoA reductase (ECR). VLCFAs are also components of seed storage triacylglycerols and sphingolipids; in yeast and mammalian cells, the latter function as signaling molecules controlling cell proliferation, stress response, and programmed cell death [8].

*Arabidopsis* mutants with defects in VLCFA synthesis display cuticular deformation, leading to alteration of pathogen-plant interactions [9], post-embryonic organ fusion [10],[11], and retardation of plant growth with abnormal morphology [12]. *PASTICCINO2* (*PAS2*) is the *Arabidopsis* gene encoding HCD, one of the enzymes involved in VLCFA synthesis [13]. A loss-of-function mutant of *PAS2* displays embryo lethality, and the leaky mutant *pas2-1*, which contains reduced amounts of VLCFAs, cuticular wax, and sphingolipids, exhibits severe morphological defects [13]–[17]. However, cuticular deformation cannot explain all of these phenotypes; in particular, the cause of defective overall growth is not well understood. Here we show that VLCFA synthesis in the epidermis is essential for plant growth, and that it suppresses cell proliferation by targeting cytokinin biosynthesis in the vasculature, thus fine-tuning cell division activity in determinate growth. Our results suggest that the epidermis sends non-autonomous signals to the vasculature and suppresses overproliferation.

## Results

### VLCFA Synthesis in the Epidermis Is Essential and Sufficient for Proper Development

**Figure 1:**
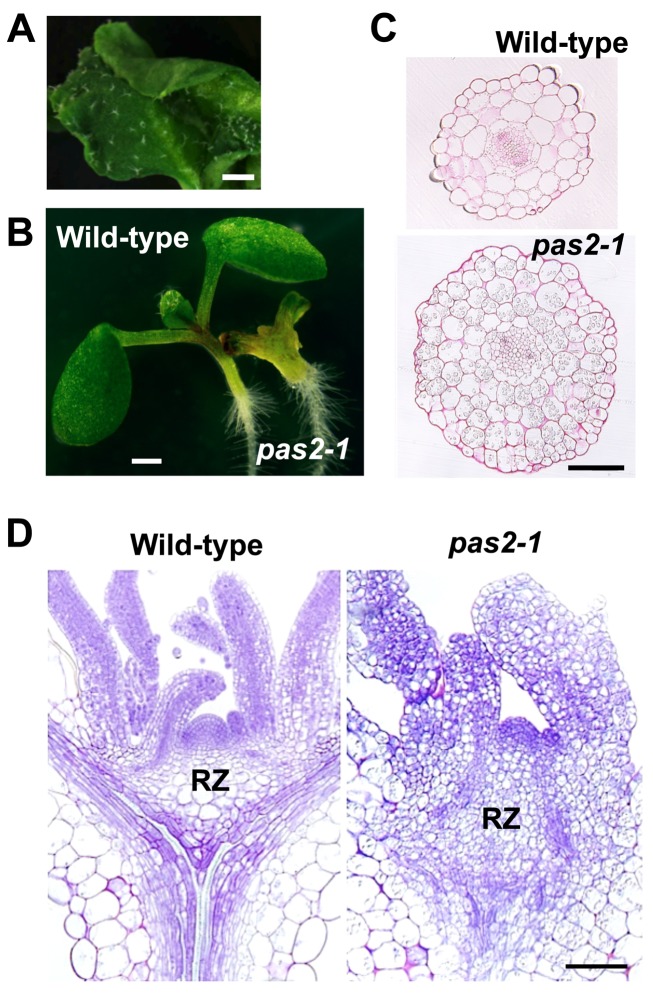
Overproliferation phenotypes of *pas2-1* mutants. (A) True leaf of a 2-wk-old *pas2-1* mutant seedling. (B) 5-d-old wild-type and *pas2-1* seedlings. (C) Cross sections of 5-d-old hypocotyls. (D) Transverse sections of shoot apices of 7-d-old seedlings. Bars, 1 mm (A), 500 µm (B), and 100 µm (C, D).

*pas2-1* mutant seedlings exhibit various morphological defects to a variable extent in individual plants; for example, true leaves are fused (Figure 1A) [14],[15],[17], and hypocotyls are swollen and possess more cortical cell layers (Figure 1B and 1C) [14]. Previous reports also noted that mutant leaves sometimes produce a callus-like structure as a result of increased cell proliferation [14]–[17]. We therefore observed the SAM, which gives rise to organs like leaves and flowers. We found that more cells accumulate in the rib zone (RZ)—a region below the self-renewing stem cell pool that contributes to the meristem pith—and that the vasculature was disorganized (Figure 1D).

**Figure 2:**
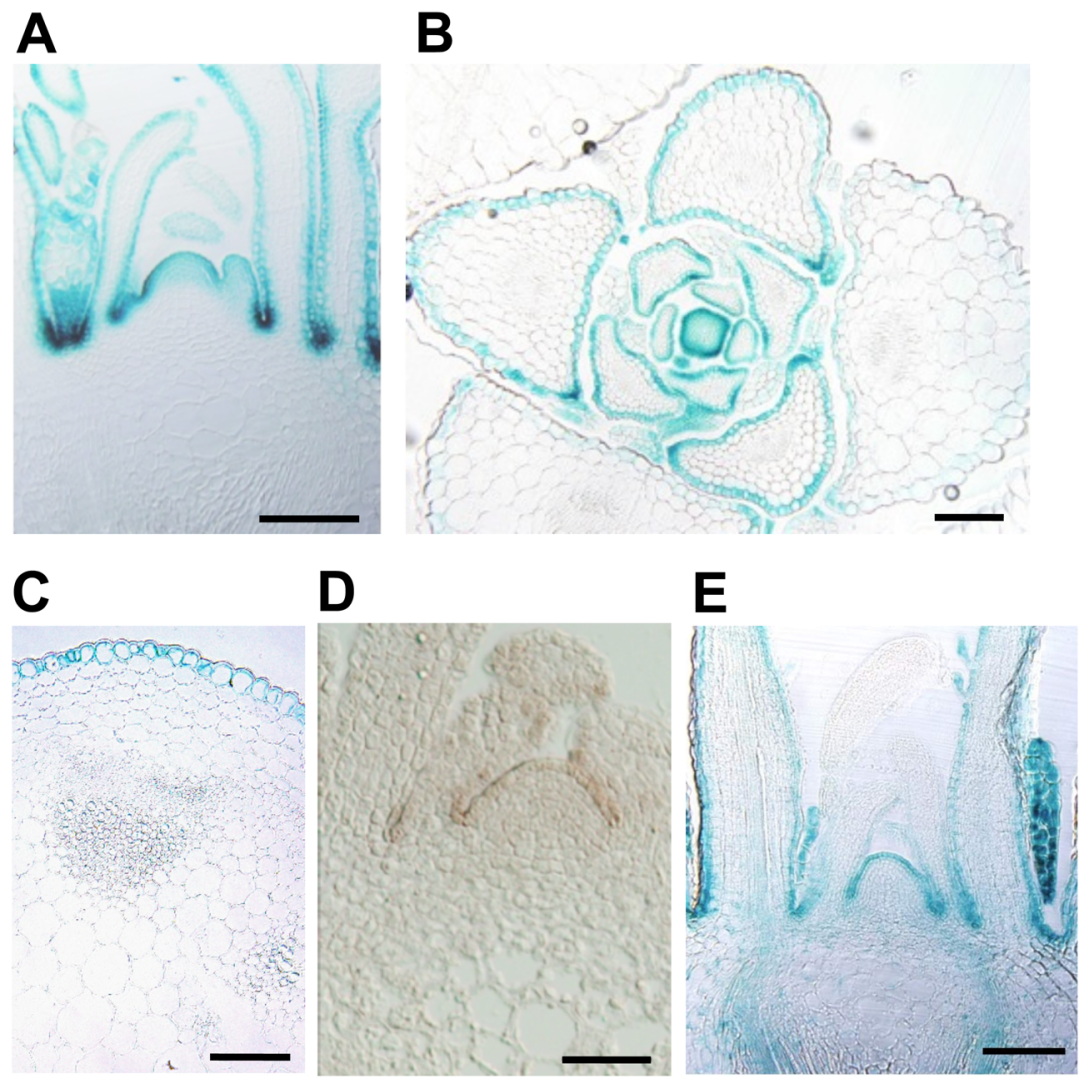
Epidermis-specific expression of *PAS2*. (A–C) GUS staining of transgenic plants carrying *ProPAS2:GUS*. Transverse section of the shoot apex of a 5-d-old seedling (A), and cross section of the shoot apex of a 10-d-old seedling (B) and inflorescence stem of a 3-wk-old seedling (C). (D) In situ hybridization of *PAS2*. A transverse section of the shoot apex of a 7-d-old wild-type seedling was hybridized with a *PAS2* antisense probe. (E) Expression pattern of *ProPAS2:PAS2–GUS* in the shoot apex of a 5-d-old seedling. Bars, 100 µm.

We monitored the expression pattern of *PAS2* using the *ProPAS2:β-glucuronidase* (*GUS*) reporter gene (a fusion of the ∼2.0-kb *PAS2* promoter and the *GUS* gene). GUS signal was detected in mature embryos, cotyledons and true leaves of seedlings, the inflorescence stem, and pistils and anthers of flowers (Figure S1). In tissue sections, *GUS* expression was observed only in the L1 layer of the SAM and in the epidermis of young leaves and the inflorescence stem (Figure 2A–2C). In situ RNA hybridization also indicated L1-specific expression (Figure 2D). To examine protein-level expression, we generated the *ProPAS2:PAS2–GUS* reporter gene (the same promoter and the full-length *PAS2* coding region fused in-frame to *GUS*); the functionality of the PAS2–GUS fusion protein was tested as described below. *GUS* expression was again detected in the L1 layer, and faint expression was noted in the vasculature (Figure 2E).

**Figure 3:**
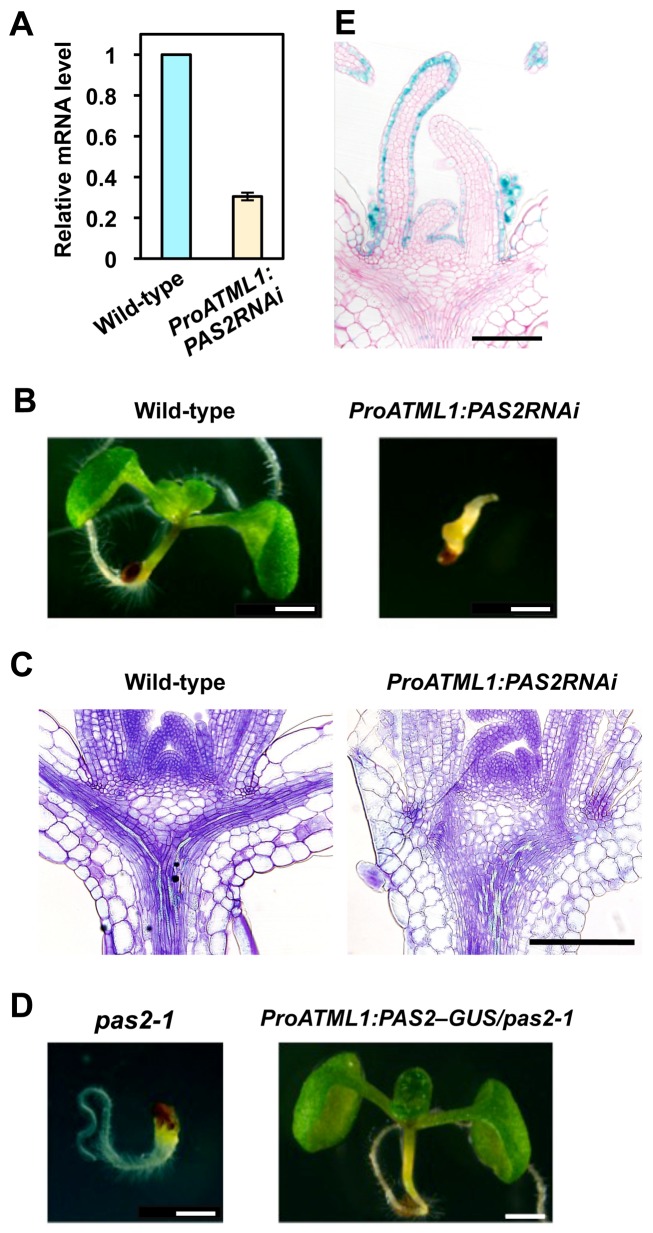
*PAS2* expression in the epidermis is essential for plant development. (A) Quantification of *PAS2* expression levels in 5-d-old seedlings. The mRNA levels were normalized to *TUBULIN4*. The expression level in wild-type expressing *ProATML1:PAS2RNAi* is indicated as a relative value, with that in wild-type set to 1. Data are presented as mean ± SD (*n* = 3). (B) 5-d-old seedlings of wild-type and wild-type expressing *ProATML1:PAS2RNAi* with a severe phenotype. Transverse sections of shoot apices of 7-d-old seedlings are shown in (C). (D) 5-d-old seedlings of *pas2-1* and *pas2-1* expressing *ProATML1:PAS2–GUS*. (E) Transverse section of the shoot apex of a 5-d-old *pas2-1* seedling expressing *ProATML1:PAS2–GUS*. Bars, 1 mm (B, D), 200 µm (C), and 100 µm (E).

To test whether *PAS2* expression in the epidermis is necessary and sufficient for normal plant development, we downregulated *PAS2* by RNAi using the *ATML1* promoter, which drives L1-specific expression [18]. The expression level of *PAS2* was reduced in the transgenic plants compared to wild-type (Figure 3A). Although the phenotypes were highly variable, we could find *pas2-1*-like phenotypes in eight of the 44 transgenic lines, such as swollen hypocotyls, fused leaves, and retarded growth (Figure 3B). Moreover, in transgenic lines showing no macroscopic phenotype, we observed overproliferation of vasculature cells and enlargement of the RZ, the latter of which appeared to be mainly due to enhanced cell expansion (Figure 3C). As mentioned above, the *ProPAS2:PAS2–GUS* reporter gene showed faint expression in the vasculature; thus, to verify that *PAS2* expression in the epidermis is sufficient for proper development, we introduced the RNAi construct under the procambial *ATHB8* promoter [19]. As a result, no *pas2-1*-like phenotype was found among 79 transgenic lines, supporting the epidermis-specific role of *PAS2* in plant development. We then introduced *ProATML1:PAS2–GUS* into the *pas2-1* mutant. GUS expression was specifically observed in the L1 layer (Figure 3E), and five of the six transgenic lines displayed fully rescued phenotypes (Figure 3D), indicating the functionality of PAS2–GUS. On the other hand, when *ProATHB8:PAS2–GUS* was introduced into *pas2-1*, none of the 19 homozygous mutants were rescued. These results demonstrate that VLCFA synthesis in the epidermis is essential and sufficient for proper plant development.

### Mild Reduction of VLCFA Content Enhances Cell Proliferation and Promotes Shoot Growth

In *pas2-1*, the cuticular wax content is severely reduced [13]; it is thus difficult to distinguish the outcome of defective cuticular formation from other effects arising from low VLCFA content. Therefore, we examined dose-dependent phenotypes in wild-type seedlings using the synthetic inhibitor cafenstrole, which blocks the first step of VLCFA elongation reactions by targeting KCS [20]. Our recent study showed that cafenstrole treatment of *Arabidopsis* seedlings reduced the content of C22 and C24 fatty acids, although our experimental conditions did not allow us to detect C26 or longer fatty acids [21].

**Figure 4:**
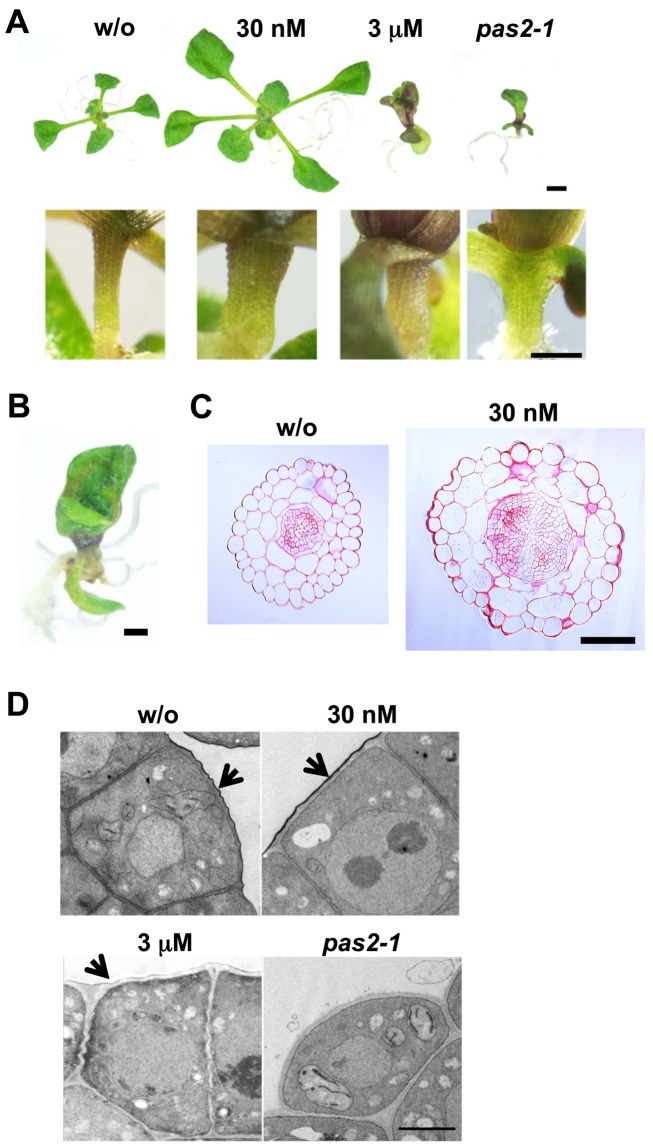
Phenotypes of cafenstrole-treated seedlings. (A) 12-d-old wild-type seedlings grown in the absence (w/o) or presence of cafenstrole (30 nM or 3 µM). *pas2-1* grown in the absence of cafenstrole is shown for comparison. Lower images show the upper region of hypocotyls. (B) Magnified view of a true leaf of 2-wk-old wild-type seedlings grown in the presence of 3 µM cafenstrole. (C) Cross sections of 7-d-old hypocotyls grown in the absence (w/o) or presence of 30 nM cafenstrole. (D) Transmission electron microscopy analysis of the L1 layer of the SAM. 3-d-old wild-type seedlings grown in the absence (w/o) or presence of 30 nM or 3 µM cafenstrole were observed. Note that, in *pas2-1*, the L1 layer is not covered with cuticular wax. Arrows indicate the electron-dense cuticular layer. Bars, 2 mm (A, upper panel), 500 µm (A, lower panel), 1 mm (B), 100 µm (C), and 2 µm (D).

**Figure 5:**
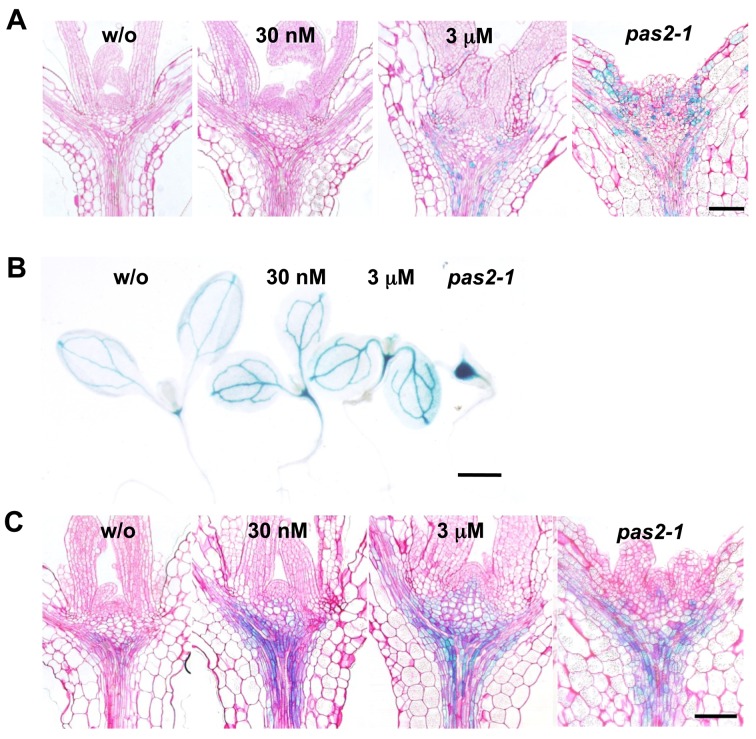
Reduced VLCFA synthesis increases the expression of *CDKB2;1* and *ARR6*. (A) Transverse sections of shoot apices of 5-d-old seedlings expressing *ProCDKB2;1:NT–GUS*. *pas2-1* grown in the absence of cafenstrole is shown for comparison. (B, C) Expression patterns of *ProARR6:GUS*. Aerial parts of 5-d-old seedlings grown in the absence (w/o) or presence of cafenstrole (30 nM or 3 µM) (B) and transverse sections of shoot apices (C). Bars, 100 µm (A, C), and 1 mm (B).

Seedlings treated with 3 µM cafenstrole displayed severe growth retardation with swollen hypocotyls and fused leaves, as observed in *pas2-1* (Figure 4A and 4B). Those treated with 30 nM cafenstrole did not show growth inhibition, but instead produced larger leaves with thicker hypocotyls (Figure 4A). Measurements of leaf area and cell size showed that, in 12-d-old seedlings, leaf blade area increased 1.7-fold after 30 nM cafenstrole treatment (14.4±2.5 mm^2^ for the control and 24.0±5.0 mm^2^ for the cafenstrole treatment; mean ± standard deviation [SD], n≥11), whereas cell size did not change significantly (830±55 µm^2^ for the control and 852±158 µm^2^ for the cafenstrole treatment) (Figure S2). Cell number also increased 1.7-fold in cafenstrole-treated leaves (17,265±2,467 for the control and 29,297±6,765 for the cafenstrole treatment) (Figure S2), accounting for the 1.7-fold enlargement of leaf blades. In shoot apices, 30 nM cafenstrole caused more cells to accumulate in the vasculature (Figure 5A), and, as a result, cell number in the vasculature of the hypocotyl dramatically increased (Figure 4C). To examine cell division activity, we used the *ProCDKB2;1:NT–GUS* reporter (comprising the *CDKB2;1* promoter and the first *CDKB2;1* exon [*NT*] fused in-frame to *GUS*), which monitors mitotic cells during the G2 and M phases [22]. The number of GUS-stained cells increased when the cafenstrole concentration was elevated, especially in the region along the vasculature (Figure 5A). These results demonstrate that a mild reduction of VLCFA content (with 30 nM cafenstrole or in *PAS2* RNAi plants) enhances proliferation of vasculature cells, while a severe reduction (with 3 µM cafenstrole or in *pas2-1*) causes overall growth retardation with impaired cuticular formation, as described below.

Transmission electron microscopy revealed that an electron-dense cuticular layer disappeared in *pas2-1*, and that only a trace of cuticular layer was formed in wild-type seedlings treated with 3 µM cafenstrole (Figure 4D). On the other hand, a thicker cuticle was formed in the presence of 30 nM cafenstrole (Figure 4D); thus, it is unlikely that cell proliferation was enhanced as a consequence of reduced cuticle synthesis. This idea is supported by the observation that *Arabidopsis* mutants specifically impaired in cuticular formation did not display enhanced cell proliferation, as described later. Moreover, the expression of the L1-specific reporter *ProPDF1:GUS* [23] retained its L1 specificity in *pas2-1* (Figure S3A), indicating that epidermal identity is maintained under low-VLCFA conditions.

### Low VLCFA Content Increases Cytokinin Level and Enhances Cell Proliferation

**Figure 6:**
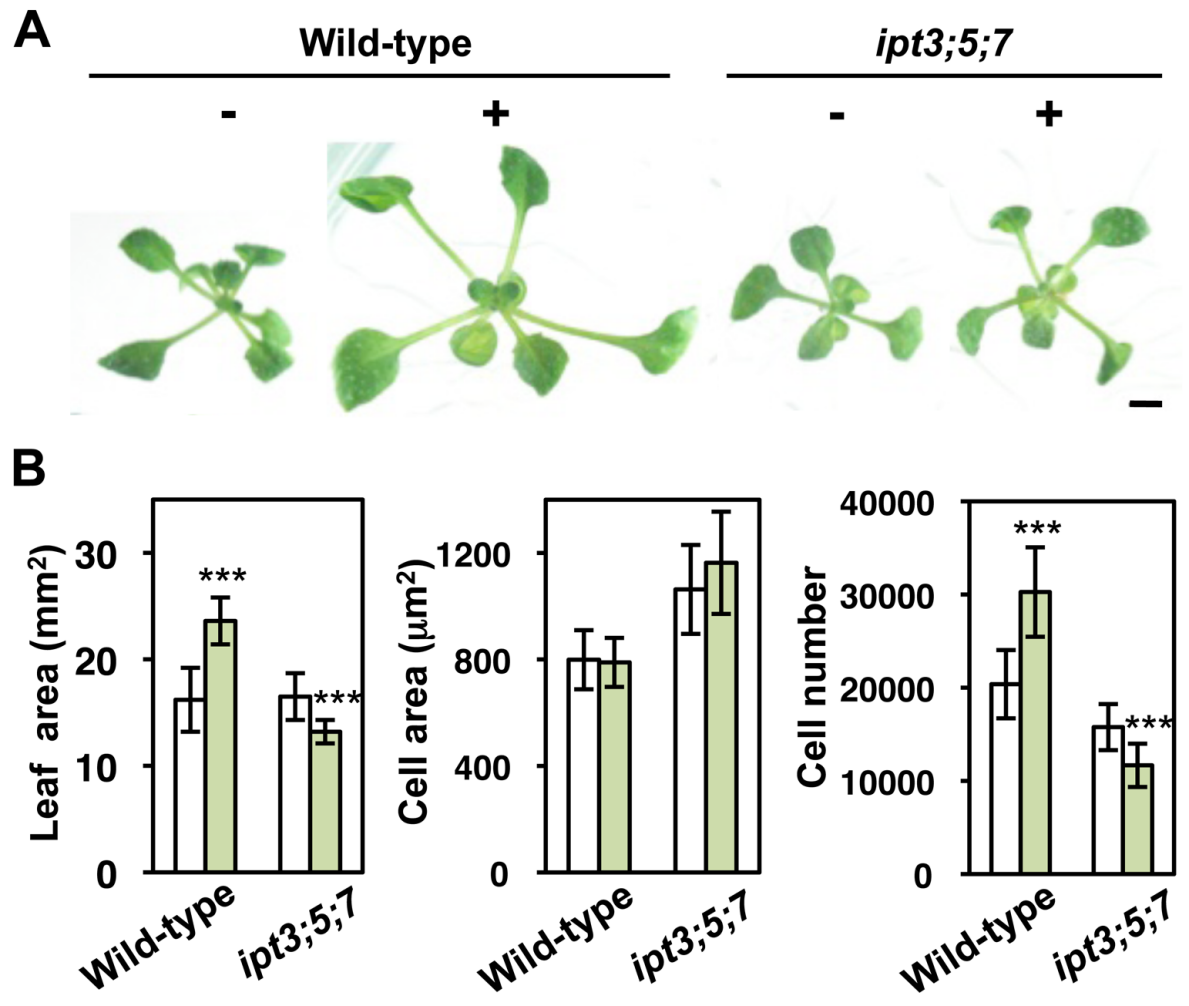
Cafenstrole-induced leaf expansion is suppressed in the *ipt3;5;7* mutant. (A) 11-d-old seedlings of wild-type and the *ipt3;5;7* triple mutant grown in the absence (−) or presence (+) of 30 nM cafenstrole. Bar, 5 mm. (B) First leaves of 11-d-old seedlings grown in the absence (white bars) or presence (green bars) of 30 nM cafenstrole were measured for leaf blade area, cell area, and cell number. Data are presented as mean ± SD (*n*≥13). Significant differences between non-treatment and 30 nM cafenstrole treatment were determined by Student's *t*-tests: ***, *p*<0.001; the other differences are not significant (*p*>0.05).

We quantified phytohormone content, and found that levels of the cytokinins isopentenyladenine (iP) and *trans*-zeatin (tZ), and of their ribosylated and phosphorylated precursors (iPR, iPRPs, tZR, and tZRPs), increased in *pas2-1* and in wild-type treated with 30 nM or 3 µM cafenstrole (Table 1). This indicates that active cytokinins are highly synthesized in *pas2-1* and after cafenstrole treatment. Indeed, expression of the primary cytokinin response marker *ARABIDOPSIS RESPONSE REGULATOR 6* (*ARR6*) [24] was stimulated by cafenstrole treatment in vascular bundles (Figure 5B and 5C). Moreover, 30 nM cafenstrole did not enlarge leaves, but instead slightly reduced the cell number and the leaf size, in *ipt3;5;7* triple mutants, in which cytokinin levels are severely decreased because of defects in cytokinin biosynthetic isopentenyltransferases (Figure 6) [25]. This finding suggests that cytokinin is associated with the cafenstrole-induced activation of cell division. A higher level of cytokinin would also explain the previously observed hypersensitivity of *pas2-1* to cytokinin treatment [14],[17]. On the other hand, the content of indoleacetic acid (IAA) and gibberellins (GA1 and GA4) did not increase, except that IAA became elevated in the presence of 3 µM (but not 30 nM) cafenstrole (Table 1).

**Table 1:** Quantification of phytohormones.

Hormone	3 DAG (pmol/g Fresh Weight)				5 DAG (pmol/g Fresh Weight)			
	w/o	30 nM	3 µM	*pas2-1*	w/o	30 nM	3 µM	*pas2-1*
**tZ**	1.08±0.22	2.22±0.07	2.31±0.25	6.36±0.85	0.81±0.22	0.95±0.10	1.44±0.24	4.49±0.24
**tZR**	2.50±0.36	36.69±2.68	29.17±0.97	118.64±18.09	1.63±0.38	6.49±1.17	19.66±2.07	108.23±15.53
**tZRPs**	33.64±4.72	221.40±11.07	189.47±28.32	245.46±17.34	19.33±1.81	71.06±9.71	143.57±7.89	363.67±31.70
**iP**	0.79±0.18	2.38±0.15	2.47±0.20	1.76±0.31	0.57±0.09	1.44±0.23	2.85±0.34	1.70±0.03
**iPR**	0.25±0.03	1.76±0.11	1.45±0.06	2.14±0.24	0.17±0.01	0.74±0.08	1.78±0.15	3.13±0.59
**iPRPs**	41.81±5.18	353.11±25.27	275.74±35.66	74.99±4.83	24.33±1.22	147.64±11.40	390.17±49.42	155.17±14.60
**IAA**	612.0±104.3	741.7±100.0	909.9±89.2	481.6±283.8	731.3±72.1	780.0±94.6	1,546.4±273.0	624.9±115.5
**GA1**	n.d.	n.d.	n.d.	n.d.	n.d.	n.d.	n.d.	n.d.
**GA4**	0.79±0.14	n.d.	n.d.	n.d.	n.d.	n.d.	n.d.	n.d.

Amounts of phytohormones were measured in 3- and 5-d-old wild-type seedlings grown in the absence (w/o) or presence of cafenstrole (30 nM or 3 µM). *pas2-1* was grown without cafenstrole. Data are presented as mean ± SD (*n*≥3).

DAG, days after germination; IAA, indoleacetic acid; n.d., not detected.

**Figure 7:**
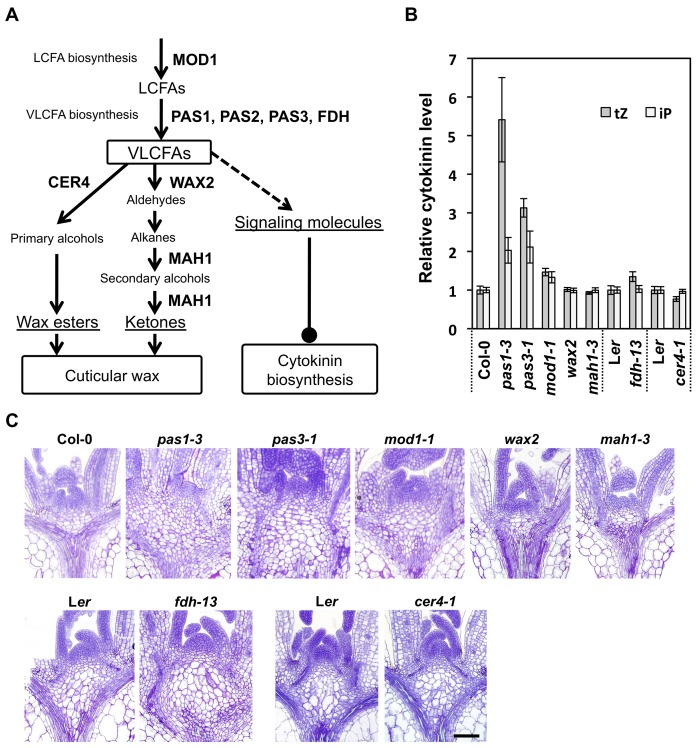
Cytokinin content and cell proliferation in LCFA- and VLCFA-related mutants. (A) Biosynthetic pathways for producing VLCFAs and cuticular wax. Enzymes and regulatory factors associated with each step are indicated. (B) Cytokinin content in various mutants. Amounts of tZ and iP were measured in 7-d-old whole seedlings, while 14-d-old seedlings were used for *fdh-13* due to phenotype-dependent identification of homozygous plants in the segregating generation. The tZ and iP levels are indicated as relative values, with those in wild-type (L*er* for *cer4-1* and *fdh-13*, and Col-0 for the others) set to 1. Data are presented as mean ± SD (*n* = 3). (C) Transverse sections of shoot apices of 7-d-old seedlings. *fdh-13* and its control (L*er*) were observed with 10-d-old seedlings. Bar, 100 µm.

To further examine whether low VLCFA content is responsible for higher cytokinin level and enhanced cell proliferation, we next used *Arabidopsis* mutants with defects in LCFA and VLCFA synthesis (Figure 7A). In mutants of *PAS3* and *PAS1*, which encode acetyl-CoA carboxylase and a scaffold protein for the elongase complex, respectively, VLCFA content is dramatically reduced and, as a result, organ growth is severely inhibited [14],[26]–[28]. As observed in *pas2-1*, these mutants contained higher amounts of tZ and iP compared to wild-type, and more cells accumulated in the RZ (Figure 7B and 7C), indicating an enhancement of cell division. A recent report demonstrated that *glossyhead1* (*gsd1*), another mutant allele for *PAS3*, did not show severe growth inhibition [29]. However, overproliferation of vasculature cells was observed in the shoot apex of *gsd1*, as in the case of 30 nM cafenstrole treatment and *PAS2* RNAi plants (Figure S4). *FIDDLEHEAD* (*FDH*)*/KCS10* encodes one of the 21 KCSs in *Arabidopsis*, and is thus associated with VLCFA synthesis; however, in the *fdh-13* mutant [2], only a mild leaf phenotype and a small reduction in C24 fatty acids were reported, probably due to redundancy in *KCS* genes [30]. Correspondingly, we detected a small increase of tZ level in seedlings, and a mild enhancement of cell proliferation in the RZ, but these phenotypes were less prominent than those in *pas* mutants (Figure 7B and 7C). Note that the RZ in the control (L*er*) was already larger than that in Col-0 (Figure 7C). We also found a similar trend in the leaky *mosaic death1* (*mod1-1*) mutant, in which the activity of the LCFA-synthesizing enzyme enoyl-ACP reductase was reduced by half (Figure 7A) [31]. Although LCFA and VLCFA content in *mod1-1* have not been reported so far, we noticed that tZ and iP levels increased slightly and that cell proliferation was enhanced in the RZ (Figure 7B and 7C). This suggests that VLCFA content might be reduced as a result of decreased LCFA synthesis, but not as severely as in *pas* mutants, leading to modest effects on cytokinin level and cell division. The above results indicate that VLCFA synthesis in the epidermis is responsible for suppressing cytokinin biosynthesis and cell proliferation. We also observed three mutants with defects in cuticular wax formation from VLCFAs, *cer4-1*, *wax2*, and *mah1-3*, which display impaired synthesis of primary alcohols, aldehydes, and secondary alcohols and ketones, respectively (Figure 7A) [11],[32],[33]. However, we found neither an increase of cytokinin level nor an enhancement of cell proliferation in these mutants (Figure 7B and 7C).

### VLCFA Synthesis Is Required for Suppression of Overproliferation by Repressing Cytokinin Biosynthesis in the Vasculature

To identify the cause of higher cytokinin production under low-VLCFA conditions, we conducted microarray analyses and examined the expression levels of cytokinin biosynthesis genes. (Microarray data have been deposited in the ArrayExpress database under accession number E-MEXP-3315.) In *pas2-1*, the mRNA levels of *IPT3* and *CYP735A2* were 3.9- and 6.6-fold higher, respectively, than in wild-type (Table S1). *IPT3* encodes one of the nine adenosine phosphate-isopentenyltransferases (IPTs), which catalyze the first and rate-limiting step of cytokinin biosynthesis to produce isopentenyladenine riboside phosphates (iPRPs) [34]. CYP735A2 converts iPRPs to *trans*-zeatin riboside phosphates (tZRPs) [35].

We examined cytokinin levels and *IPT3* expression in the *pas2-1* mutant carrying *ProATML1:PAS2–GUS*, which rescued *pas2-1* phenotypes (Figure 3D). As described above, levels of tZ and iP were highly elevated in *pas2-1* compared to those in wild-type (Table 1), but in *pas2-1* carrying *ProATML1:PAS2–GUS*, no such increase of cytokinin content was detected (tZ, 0.57±0.06 pmol/g fresh weight for Col-0 and 0.71±0.13 pmol/g for the transgenic line; iP, 0.49±0.03 pmol/g for Col-0 and 0.51±0.03 pmol/g for the transgenic line; mean ± SD, 7-d-old seedlings [*n* = 3]). The elevated level of *IPT3* transcripts in *pas2-1* was also reduced to the wild-type level by *PAS2–GUS* expression in the epidermis (the relative mRNA level, with that for wild-type set to 1, was 3.77±0.15 for *pas2-1* and 0.81±0.09 for *pas2-1* carrying *ProATML1:PAS2–GUS*; mean ± SD, 7-d-old seedlings [*n* = 3]). These results indicate that VLCFA synthesis in the epidermis is required to suppress not only cytokinin biosynthesis but also *IPT3* expression.

**Figure 8:**
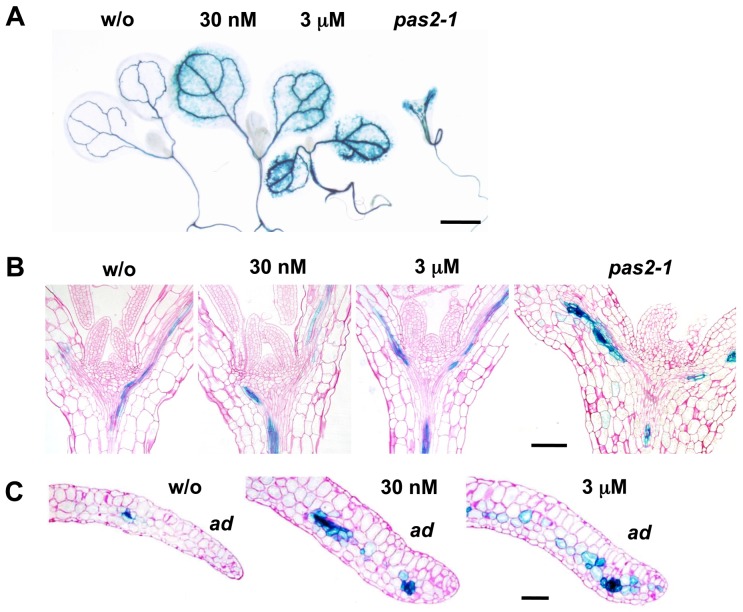
Reduced VLCFA synthesis increases the expression of *IPT3*. Expression patterns of *ProIPT3:GUS*. Aerial parts of 5-d-old seedlings grown in the absence or presence of cafenstrole (A), and transverse sections of shoot apices (B) and cotyledons (C). *pas2-1* grown in the absence of cafenstrole is shown for comparison. *ad*, adaxial side of cotyledons. Bars, 1 mm (A) and 100 µm (B, C).

We then monitored *IPT3* expression in 5-d-old seedlings using the *promoter:GUS* reporter. Consistent with a previous observation of *IPT3* expression in the phloem [19], we detected the GUS signal in vascular bundles (Figure 8). Cafenstrole treatment increased the intensity of the GUS signal and extended the expression domain in shoot apices and leaves; a similar expression pattern was also observed in *pas2-1* (Figure 8A and 8B). In cafenstrole-treated leaves, expression of the procambial marker *ProATHB8:GUS* [21] was restricted to vascular bundles (Figure S3B and S3C), but *IPT3* expression extended to spongy mesophyll cells (Figure 8C). This indicates that low-VLCFA conditions increase *IPT3* expression in the vasculature and cause ectopic expression in non-vascular cells.

To examine whether increased cytokinin synthesis is a cause or a consequence of the overproliferation phenotype, we monitored *IPT3* expression after transfer of 3-d-old seedlings to a medium containing 30 nM or 3 µM cafenstrole. We also observed the *ProCYCB1;2:NT–GUS* reporter (comprising the *CYCB1;2* promoter and the N-terminal region of *CYCB1;2* [*NT*] fused in-frame to *GUS*), which monitors G2/M phase cells [36]. As shown in Figure S5, higher *IPT3* expression was noted after 6 h and 12 h for 3 µM and 30 nM cafenstrole, respectively, compared with the non-treated control. By contrast, *CYCB1;2* expression increased from 12 to 24 h in the SAM and in young true leaves regardless of cafenstrole treatment (Figure S5), suggesting a general activation of cell division at this developmental stage. Expression was even higher after 48 h for both 30 nM and 3 µM cafenstrole, but not for the control (Figure S5), demonstrating that cafenstrole-induced overproliferation occurred later than 24 h. Measurement of cytokinin content revealed that cytokinin precursors, especially iPR and iPRPs, increased after 6 to 12 h of 3 µM cafenstrole treatment, and that iP and tZ increased after 24 h (Table S2). These results indicate that cafenstrole induces cytokinin synthesis, which is then followed by activation of cell division, implying that enhanced cytokinin synthesis is the cause of overproliferation triggered by low-VLCFA conditions.

**Figure 9:**
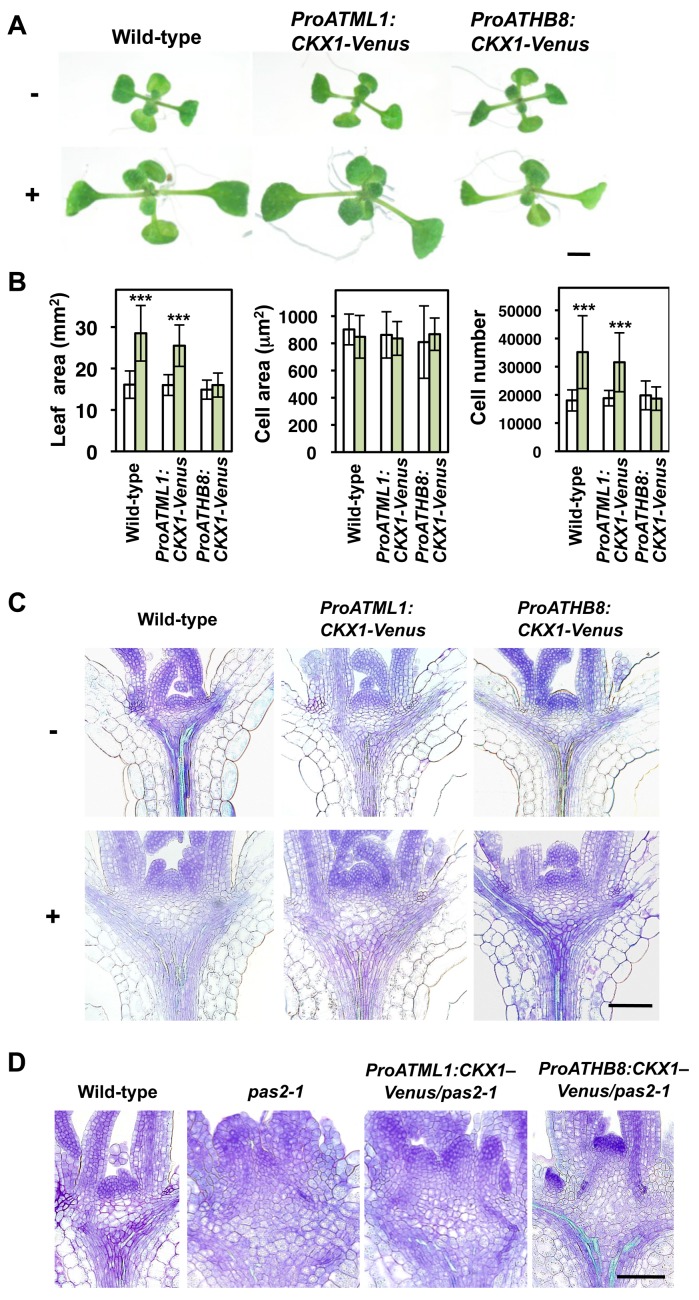
Vasculature-specific expression of *CKX1* suppresses cafenstrole-induced enhancement of plant growth. (A) 8-d-old seedlings of wild-type, *ProATML1:CKX1–Venus* and *ProATHB8:CKX1–Venus* grown in the absence (−) or presence (+) of 30 nM cafenstrole. (B) First leaves of 12-d-old seedlings of wild-type, *ProATML1:CKX1–Venus* and *ProATHB8:CKX1–Venus* grown in the absence (white bars) or presence (green bars) of 30 nM cafenstrole were measured for leaf blade area, cell area and cell number. Data are presented as mean ± SD (*n*≥11). Significant differences between non-treatment and 30 nM cafenstrole treatment were determined by Student's *t*-tests: ***, *p*<0.001; the other differences are not significant (*p*>0.05). (C, D) Transverse sections of shoot apices. 5-d-old seedlings of wild-type, *ProATML1:CKX1–Venus* and *ProATHB8:CKX1–Venus* grown in the absence (−) or presence (+) of 30 nM cafenstrole (C), and 7-d-old seedlings of wild-type, *pas2-1*, and *pas2-1* expressing *ProATML1:CKX1–Venus* or *ProATHB8:CKX1–Venus* (D). *CKX1–Venus* was introduced into the heterozygous *pas2-1* mutant, and homozygous plants were isolated for observation of shoot apices. Bars, 5 mm (A) and 100 µm (C, D).

The above results suggested an interesting hypothesis, namely, that VLCFA synthesis in the epidermis is required to confine cytokinin biosynthesis to the vasculature and prevent cells from overproliferating. To test this hypothesis, we examined whether the effect of cafenstrole is suppressed by reducing cytokinin levels. We expressed the gene for Venus-fused cytokinin oxidase 1 (CKX1), which degrades active forms of cytokinins [37], under the control of *ATML1* and *ATHB8* promoters. Venus fluorescence showed that the *ATML1* and *ATHB8* promoters conferred epidermis- and vasculature-specific expression, respectively (Figure S6A and S6B). We measured leaf area in four independent lines for each promoter construct, and found that 30 nM cafenstrole enlarged leaves in wild-type and *ProATML1:CKX1–Venus*, but that no such enlargement occurred in *ProATHB8:CKX1–Venus* (Figures 9A, 9B, and S6C). The latter effect was due to the suppression of cafenstrole-induced enhancement of cell proliferation in leaves (Figure 9B). Enhanced cell accumulation in the vasculature, expansion of hypocotyl width at the base of cotyledons, and an increase in vascular cell number in hypocotyls were also suppressed by *CKX1–Venus* expression in vascular bundles (Figures 9C, S6D, and S6E). We also expressed *CKX1–Venus* in *pas2-1*, but the macroscopic phenotype of the mutant was not suppressed with either promoter, probably owing to severely impaired cuticular formation. However, when shoot apices were observed microscopically, we found that the enhanced cell accumulation in the RZ and disorganization of the vasculature were partially suppressed with *ProATHB8:CKX1–Venus*, but not with *ProATML1:CKX1–Venus* in *pas2-1* (Figure 9D). These results indicate that, under low-VLCFA conditions, an increase of cytokinin biosynthesis in the vasculature is the major cause of overproliferation. The inability of *ProATML1:CKX1–Venus* to suppress overproliferation suggests that non-cell-autonomous factors (other than cytokinins) act from mesophyll cells to the epidermis to promote cell division, as reported previously [38].

## Discussion

In this study, we showed that a higher concentration of cafenstrole (3 µM) caused severe growth defects, notably swollen hypocotyls and fused leaves, which are similar to those observed in the leaky *pas2-1* mutant. More cells accumulated in the RZ of *pas2-1*, and the number of cortical cell layers increased in the hypocotyl. In contrast, at a lower concentration (30 nM), seedlings showed neither overall growth inhibition nor organ fusions; rather, the leaves were enlarged due to increased cell number. Enhanced cell proliferation was also observed in the vasculature in shoot apices, resulting in a dramatic increase of cell number in the vasculature of hypocotyls. When *PAS2* expression was specifically downregulated in the epidermis, we could again observe disorganized vasculature due to enhanced cell proliferation. It is likely that, in *pas2-1* and in wild-type plants treated with 3 µM cafenstrole, the stimulatory effect on cell division might be difficult to observe macroscopically, except for the swollen hypocotyl, due to impaired cuticular formation and consequent growth defects. However, a common feature was observed following mild or severe inhibition of VLCFA synthesis, namely, enhanced cell proliferation in the vasculature or in the RZ, respectively. It is noteworthy that, in *pas2-1*, cell accumulation was prominent in the RZ but not in the vasculature. One possible explanation for this observation is that the amount of cytokinin in the vasculature may be so high that cell division is actually inhibited. The lower level of cytokinin in the RZ than in the vasculature may efficiently enhance cell proliferation. It is also probable that faster accumulation of RZ cells in *pas2-1* suppresses cell division in the vasculature by intertissue communication. Further studies are needed to examine such possibilities.

PAS2 has been identified as an antiphosphatase, which interacts with tyrosine-phosphorylated cyclin-dependent kinase A (CDKA) and prevents it from being dephosphorylated and activated [39]. However, in a *pas2* mutant carrying phosphomimic mutations in *CDKA*, the phenotype of phosphomimic *CDKA* plants was not epistatic to the *pas2* phenotype; rather, the two phenotypes were additive, indicating that PAS2 functions in parallel to CDKA [40]. This is supported by the fact that PAS2 is exclusively localized to the endoplasmic reticulum, whereas CDKA is distributed in both the nucleus and the cytoplasm [13],[41]. Here, we observed enhanced cell proliferation in the RZ or in the vasculature not only in *pas2*, but also in other VLCFA-related mutants, such as *pas1*, *pas3*, *gsd1*, and *fdh*, as well as by treatment with the KCS inhibitor cafenstrole. Therefore, the overproliferation phenotype is not specifically linked to PAS2 functions, but instead is caused by inhibition of VLCFA synthesis.

**Figure 10:**
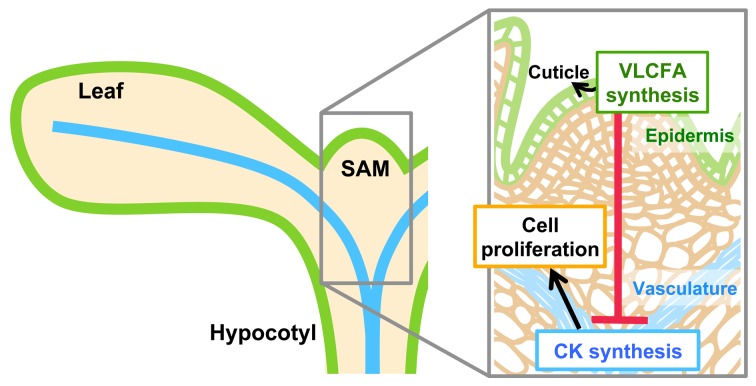
A model for restriction of cell proliferation by VLCFA synthesis. VLCFA synthesis in the epidermis confines cytokinin (CK) synthesis to the vasculature via non-autonomous signals, and restricts cell proliferation. VLCFA is also used for the synthesis of cuticular wax; thus, cuticle formation and cell proliferation are coordinately controlled by VLCFA synthesis during shoot development. Green and blue lines represent the epidermis and the vasculature, respectively.

We revealed that, in *pas2-1* and under cafenstrole treatment, *IPT3* expression in the vasculature was elevated and its domain expanded to the spongy mesophyll cells. Indeed, the content of active cytokinins increased prior to the activation of cell division, whereas the overproliferation phenotype was suppressed in *ipt3;5;7* mutants and by vasculature-specific degradation of cytokinins. These results demonstrate that VLCFA synthesis in the epidermis confines cytokinin biosynthesis to the vasculature and restricts cell proliferation. The idea that *IPT3* is a possible target of epidermis-derived signals is supported by a previous report that overexpression of *IPT3* in *Arabidopsis* resulted in a 3.4-fold increase of cytokinin content, and enlarged leaves with increased cell number [42]. However, it is also likely that *CYP735A2*, whose expression increased 6.6-fold in *pas2-1*, is another target. In *Arabidopsis* and rice plants, impairment of VLCFA synthesis elevates the expression of *KNOTTED-like homeobox* (*KNOX*) genes [17],[43]. Moreover, overexpression of class I *KNOX* (*KNOXI*) genes is known to promote cytokinin synthesis in *Arabidopsis* [44]. However, it is unlikely that VLCFA synthesis in the epidermis restricts the cytokinin level by controlling *KNOXI* expression, because we observed overproliferation not only in the SAM but also in leaves, where *KNOXI* genes are not expressed. It is known that cytokinin induces expression of the *KNOXI* genes *KNAT1/BP* and *STM* [45], suggesting that *KNOX* upregulation under low-VLCFA conditions results from increased cytokinin synthesis in the shoot apex. Our results indicate that epidermis-derived signals fine-tune cell division activity in internal tissue, suggesting that shoot growth is controlled by the interaction between the surface (epidermis) and the axis (vasculature) of the plant body (Figure 10). Indeed, perturbing this regulation by lowering VLCFA synthesis increased leaf size, demonstrating that non-autonomous signals are essential to restrict organ size.

*Arabidopsis* mutants with defects in cuticular wax formation from VLCFAs (*cer4*, *mah1*, *wax2*) did not exhibit phenotypes similar to those observed in *pas* mutants or cafenstrole-treated wild-type plants. Although we cannot exclude the possibility that some level of wax classes synthesized in these mutants suppresses the overproliferation phenotype, it is more likely that VLCFA derivatives function as signaling molecules to control cytokinin biosynthesis and cell division (Figure 7A). In yeast and animals, sphingolipids made from VLCFAs act as signaling molecules controlling cell proliferation, cell death and stress responses [8]. Although *Arabidopsis* mutants defective in sphingolipid biosynthesis are impaired in cell growth, and in severe cases die [46],[47], some types of sphingolipids may control cell division by affecting cytokinin synthesis. It is also possible that VLCFA-containing lipids may function as mediators or ligands that control gene transcription, as suggested in mammals, yeast, and bacteria [48]. Indeed, arachidonic acid is known to induce stress-related gene expression and elicit defense signaling in *Arabidopsis* [49]. It is also likely that some metabolites, whose levels change depending on VLCFA synthesis, confine cytokinin biosynthesis to the vasculature.

In *Arabidopsis*, 21 genes have been identified for KCS, which catalyzes the first step of VLCFA elongation reactions. Some of them are expressed predominantly in the epidermis, as observed for *PAS2* [50]. Indeed, *PAS2*, *KCS11*, *KCS16*, and *KCS20* have one L1-box, and *KCS6*, *KCS9*, *KCS10/FDH*, and *KCS18* have two L1-boxes, in their promoter regions, at which transcription factors ATML1 and PDF2 bind and control the L1/epidermis-specific gene expression [1]. Four *kcs* mutants, *kcs6*, *kcs10/fdh*, *kcs2*, and *kcs20*, exhibit a glossy appearance and/or organ fusion, but no overproliferation phenotype was described [51]–[54]. However, we observed mildly enhanced cell proliferation in the RZ of *fdh-13*, suggesting that other *kcs* mutants may also accumulate more cells in the RZ (or in the vasculature) than wild-type plants. Alternatively, specific VLCFAs may be required to suppress cell proliferation, because the distribution of various VLCFA species may depend on the substrate preference of each KCS (e.g., for a particular carbon chain length). HCD is unlikely to have such substrate preference, as it is encoded by the single-copy gene *PAS2* in *Arabidopsis*; thus, the *pas2* mutation reduces the overall level of VLCFAs including those that are responsible for suppressing cell proliferation. Further studies will reveal which VLCFA species or VLCFA-containing lipids are associated with non-autonomous signals to suppress cell proliferation in tissues.

Previously, cyclin-dependent kinase inhibitor genes were ectopically expressed in the L1 layer, and meristem organization was investigated [55]. Cell number in the epidermis was reduced, while that in the cortex and mesophyll was the same as in wild-type. These and our present observations indicate that cell proliferation in shoot growth is not coordinated between the L1 and the inner layers; rather, it is controlled by VLCFA-derived signals that act on cytokinin biosynthesis in the vasculature. A likely benefit of this system is that plants can coordinate cuticular wax formation and organ growth, and can thus maintain proper development under various environmental conditions. Several *KCS* genes are induced by abiotic stress, such as salt, dehydration, and osmotic stress [50]; the transcription factor MYB30, which activates expression of VLCFA biosynthesis genes, is also induced by pathogen infection [56]. Therefore, plants may deploy mechanisms to actively form cuticular wax and suppress cell proliferation to minimize energy consumption under stressful conditions. It is also known that BES1, a downstream transcription factor in the brassinosteroid signaling pathway, directly interacts with and activates MYB30 [56],[57]. While BES1 is involved in generating cell growth-promoting signal(s) from the L1 to inner layers [6], brassinosteroid signaling in the epidermis may also control cell proliferation by activating MYB30 and VLCFA synthesis. Identification of non-autonomous signals will reveal how plants limit organ growth and adapt to changing environments by controlling cell growth and proliferation.

## Materials and Methods

### Plant Materials and Growth Conditions

*ProPDF1:GUS* [23] and *ProIPT3:GUS* lines [34], *pas1-3* [27], *pas2-1* [14], *pas3-1* [14], *fdh-13* [2], *mod1-1* [31], *gsd1* [29], and *ipt3;5;7* [27] were described previously. Seeds of *ProARR6:GUS* (N25262), *ProATHB8:GUS* (N296), *mah1-3* (SALK_133155), and *wax2* (SALK_020265) were obtained from the Arabidopsis Biological Resource Center. Seeds of *cer4-1* (N34) were obtained from the European Arabidopsis Stock Centre. All *Arabidopsis* plants used were in the Columbia (Col-0) background, except that *cer4-1* and *fdh-13* were in the Landsberg *erecta* (L*er*) background. To isolate the *pas2-1* mutant, a genomic DNA fragment was amplified by PCR using a set of primers (5′-TCCACTGGTATCAGGGGAG-3′ and 5′-CTACTGAGAAGGAACCAATGATT-3′), and treated with *Mva*I to observe the digestion pattern. *Arabidopsis* plants were grown in Murashige and Skoog (MS) medium (1× MS salts, 1× MS vitamins, 2% [w/v] sucrose, and 0.8% agar [pH 6.3]) under continuous light conditions at 23°C. Cafenstrole (HPLC standard grade, Wako Chemical) was dissolved in dimethylsulfoxide at appropriate concentrations, and diluted 1,000-fold into the media.

### Plasmid Construction for Plant Transformation

The 2-kb promoter fragment of *PAS2* was PCR-amplified and cloned into the *Sal*I-*Bam*HI site of the pBI101.2 binary vector (Clontech Laboratories) to generate a fusion construct with *GUS* (*ProPAS2:GUS*). The promoter and the coding region of *PAS2* were PCR-amplified from genomic DNA and cloned into the Gateway entry vector pDONR221 (Invitrogen) by a BP reaction. An LR reaction was performed with the destination vector pGWB3 [58] to generate a binary vector carrying the fusion construct with *GUS* (*ProPAS2:PAS2–GUS*). The 3.4-kb promoter fragment of *ATML1* and the coding region of *PAS2* were PCR-amplified from genomic DNA and cloned into the *Eco*RI and *Sma*I sites, respectively, of the pBluescript II KS(-) vector (Stratagene). The resultant plasmid was digested with *Bam*HI and *Hin*dIII, and the fragment was cloned into the *Hin*dIII-*Bam*HI site of the pBI101 binary vector (Clontech) to generate *ProATML1:PAS2–GUS*. To make the *PAS2* RNAi construct, the region encompassing nucleotides 6 to 641 of the *PAS2* ORF was cloned into the *Eco*RI-*Kpn*I and *Bam*HI-*Hin*dIII sites of the pHANNIBAL vector [59]. The 35S promoter region in the vector was then replaced by the 3.4-kb *ATML1* promoter, and the resultant *ProATML1:PAS2RNAi* fragment was cloned into the *Not*I site of the pART27 binary vector [60]. To express the CKX1–Venus fusion protein, the ORF of *Venus* and the genomic fragment comprising the coding region of *CKX1* were PCR-amplified and tandemly cloned into the *Sal*I site of pAN19, a derivative of the pUC19 vector (Invitrogen), to be in-frame with each other. The resultant *CKX1–Venus* construct was then PCR-amplified and cloned into pDONR221 by a BP reaction. The 3.4-kb and 1.7-kb promoter fragments of *ATML1* and *ATHB8*, respectively, were PCR-amplified and cloned into the Gateway entry vector pDONRP4-P1R (Invitrogen) by a BP reaction. An LR reaction was conducted with the destination vector pGWB501 [61] and the above-mentioned entry vectors to generate a binary vector carrying each promoter fragment fused to *CKX1–Venus*. To express *PAS2–GUS* and the *PAS2* RNAi construct under the *ATHB8* promoter, the fragments of *PAS2–GUS* and the *PAS2* RNAi construct were PCR-amplified using the above-mentioned binary vectors with the *ATML1* promoter, and cloned into pDONR221 by a BP reaction. These entry clones were used for an LR reaction with the destination vector pGWB501 and the entry vector pDONRP4-P1R carrying the *ATHB8* promoter fragment. Primers used for plasmid constructions are listed in Table S3.

### Histological Analysis

GUS staining and tissue sectioning were performed as described previously [62]. For counter-staining, sections were incubated with 0.05% (w/v) toluidine blue O. In the case of GUS-stained samples, sections were incubated with 0.05% (w/v) ruthenium red.

### In Situ RNA Hybridization

*Arabidopsis* tissues were fixed in FAA (50% [v/v] ethanol, 5% [v/v] acetic acid, and 3.7% [v/v] formaldehyde), and 8-µm paraffin sections were hybridized with digoxygenin-labeled probes according to the protocol from the manufacturer (Roche). The *PAS2* probe was the antisense strand corresponding to the region 6 to 506 of the *PAS2* ORF.

### Microscopy Observation

For measurements of leaf blade area, healthy first leaves were harvested and fixed in a solution of 2.5% glutaraldehyde, and stored at 4°C. The area of edited microscopic images was measured using the image analysis program NIH ImageJ 1.43u (http://rsb.info.nih.gov/nih-image/). To measure cell size and cell number, data were collected by scanning images of the abaxial epidermis located at 50% of the distance between the tip and the base of the leaf blade, halfway between the midrib and the leaf margin. Images that included at least 40 cells in focus were edited using Photoshop Elements 6 (Adobe, http://www.adobe.com/). Epidermal cells in the edited image were counted, and the area of the edited image was measured with ImageJ. The average cell area was determined on the basis of these measurements. The total number of epidermal cells on the abaxial side was estimated on the basis of the average cell area and leaf blade area. For detection of Venus fluorescence, plant seedlings were embedded in 7% agarose and sliced manually with a razor, and sections were observed with a confocal laser scanning microscope (LSM710; Carl Zeiss).

### Transmission Electron Microscopy

Samples were fixed with 2.5% glutaraldehyde in phosphate buffer (pH 7.0) at 4°C overnight, and then postfixed with 1% osmium tetroxide in the same buffer at 4°C for 1 h. Fixed samples were dehydrated in an ethanol series and embedded in Spurr resin, and polymerized at 73°C. Ultrathin sections were prepared with a diamond knife, stained with uranyl acetate and lead citrate, and observed with a JEOL 1200EX microscope.

### Quantification of Phytohormones

Sampling of about 100 mg of fresh whole seedlings was repeated three times. Extraction and determination of hormones were performed as described previously [63]. Data were processed by MassLynx software with QuanLynx (version 4.0, Waters).

### Microarray Analysis

Total RNA was extracted from 3-d-old whole seedlings using TRIzol (Invitrogen) and purified with an RNeasy microkit (QIAGEN) as described in the manufacturer's instructions. GeneChip analyses were independently performed twice with the *Arabidopsis* ATH1 Genome Array (Affymetrix) as described in the GeneChip Expression Analysis Technical Manual (Affymetrix). Probe synthesis was performed with the GeneChip 3′ IVT Express kit (Affymetrix) following the manufacturer's protocol. Hybridization and washes were performed as described in the GeneChip Expression Analysis Technical Manual. Signal detection and global normalization were performed using GeneChip Operating Software (Affymetrix; version 1.4) with standard parameters.

## Supporting Information

Figure S1 Expression pattern of *PAS2*. GUS staining of transgenic plants carrying *ProPAS2:GUS*. Mature embryo (A), 5-d-old seedling (B), 8-d-old seedling (C), inflorescence (D), and flowers and anthers (E). Bars, 100 µm (A), 1 mm (B, E), and 2 mm (C, D).
(TIF)

Figure S2 Kinematic analysis of leaf growth. First leaves of wild-type seedlings grown in the absence (w/o) or presence of 30 nM cafenstrole were measured for leaf blade area, cell area, and cell number per leaf. Data are presented as mean ± SD (*n*≥10). DAG, days after germination.
(TIF)

Figure S3 Reduced VLCFA synthesis does not affect the expression patterns of *PDF1* and *ATHB8*. (A) Expression pattern of *ProPDF1:GUS* in wild-type and *pas2-1*. Transverse sections of shoot apices of 5-d-old seedlings. (B, C) Expression pattern of *ProATHB8:GUS*. 5-d-old wild-type seedlings grown in the absence (w/o) or presence of cafenstrole (30 nM or 3 µM) (B) and cross sections of cotyledons (C). *pas2-1* grown in the absence of cafenstrole is shown for comparison. Bars, 50 µm (A), 1 mm (B), and 100 µm (C).
(TIF)

Figure S4 Enhanced cell proliferation in the *gsd1* mutant. Transverse sections of shoot apices of 7-d-old Col-0 and *gsd1* seedlings. Bar, 100 µm.
(TIF)

Figure S5 *IPT3* and *CYCB1;2* expression after cafenstrole treatment. 3-d-old seedlings carrying *ProIPT3:GUS* or *ProCYCB1;2:NT–GUS* were transferred onto a medium without cafenstrole (w/o), or containing 30 nM or 3 µM cafenstrole, and GUS expression was observed at the indicated time points thereafter. Enlarged images of *ProCYCB1;2:NT–GUS* seedlings after 48 h are shown below (a–c). Bars, 1 mm and 500 µm (a–c).
(TIF)

Figure S6 Vasculature-specific expression of *CKX1* suppresses the increase of hypocotyl width caused by cafenstrole treatment. (A, B) Expression patterns of *CKX1–Venus* controlled by the *ATML1* (A) and *ATHB8* (B) promoters. Transverse sections of shoot apices (left image of A, B) and cross section of a first leaf (right image of A). Venus fluorescence was merged with autofluorescence. Asterisks indicate the SAM. (C) First leaves of 10-d-old seedlings of wild-type, *ProATML1:CKX1–Venus* and *ProATHB8:CKX1–Venus* grown in the absence (white bars) or presence (green bars) of 30 nM cafenstrole were measured for leaf blade area. For each promoter construct, three independent lines, which are different from those shown in Figure 9, were used for measurement. Data are presented as mean ± SD (*n*≥20). (D) Measurement of hypocotyl width. Hypocotyls of 8-d-old seedlings grown in the absence (−) or presence (+) of 30 nM cafenstrole were measured. Data are presented as mean ± SD (*n*≥20). Significant differences between wild-type and *CKX1–Venus* transgenic seedlings were determined by Student's *t*-test: ***, *p*<0.001; the other differences are not significant (*p*>0.05). (E) Cross sections of 5-d-old hypocotyls grown in the absence (−) or presence (+) of 30 nM cafenstrole. Bars, 50 µm (A), 20 µm (B), and 100 µm (E).
(TIF)

Table S1 Expression levels of cytokinin biosynthesis genes in the *pas2-1* mutant. Average values of biological duplicates in microarray analysis are shown as relative values, with those for wild-type set to 1.
(DOCX)

Table S2 Cytokinin contents after cafenstrole treatment. 3-d-old wild-type seedlings were transferred onto a medium without cafenstrole (w/o), or containing 30 nM or 3 µM cafenstrole, and measured for cytokinin contents after indicated time points. Data are presented as mean (pmol/g fresh weight) ± SD (*n* = 3).
(DOCX)

Table S3 Primers used for plasmid constructions. CDS (coding sequence) was PCR-amplified from the genomic DNA.
(DOCX)

## Abbreviations

CDKA: cyclin-dependent kinase A
CKX: cytokinin oxidase
GUS: β-glucuronidase
HCD: 3-hydroxyacyl-CoA dehydratase
iP: isopentenyladenine
IPT: adenosine phosphate-isopentenyltransferase
KCR: 3-ketoacyl-CoA reductase
KCS: ketoacyl-CoA synthase
RZ: rib zone
SAM: shoot apical meristem
tZ: *trans*-zeatin
VLCFA: very-long-chain fatty acid

## References

[1] Abe M, Katsumata H, Komeda Y, Takahashi T (2003) Regulation of shoot epidermal cell differentiation by a pair of homeodomain proteins in *Arabidopsis* . Development 130: 635–643. 12505995 10.1242/dev.00292

[2] Tanaka T, Tanaka H, Machida C, Watanabe M, Machida Y (2004) A new method for rapid visualization of defects in leaf cuticle reveals five intrinsic patterns of surface defects in *Arabidopsis* . Plant J 37: 139–146. 14675439 10.1046/j.1365-313x.2003.01946.x

[3] Tanaka H, Watanabe M, Sasabe M, Hiroe T, Tanaka T, et al (2007) Novel receptor-like kinase ALE2 controls shoot development by specifying epidermis in *Arabidopsis* . Development 134: 1643–1652. 17376810 10.1242/dev.003533

[4] Fleming AJ, McQueen-Mason S, Mandel T, Kuhlemeier C (1997) Induction of leaf primordia by the cell wall protein expansin. Science 276: 1415–1418.

[5] Pien S, Wyrzykowska J, McQueen-Mason S, Smart C, Fleming A (2001) Local expression of expansin induces the entire process of leaf development and modifies leaf shape. Proc Natl Acad Sci U S A 98: 11812–11817. 11562463 10.1073/pnas.191380498 PMC58813

[6] Savaldi-Goldstein S, Peto C, Chory J (2007) The epidermis both drives and restricts plant shoot growth. Nature 446: 199–202. 17344852 10.1038/nature05618

[7] Kunst L, Samuels L (2009) Plant cuticles shine: advances in wax biosynthesis and export. Curr Opin Plant Biol 12: 721–727. 19864175 10.1016/j.pbi.2009.09.009

[8] Worrall D, Ng CKY, Hetherington AM (2003) Sphingolipids, new players in plant signaling. Trends Plant Sci 8: 317–320. 12878015 10.1016/S1360-1385(03)00128-6

[9] Reina-Pinto JJ, Yephremov A (2009) Surface lipids and plant defenses. Plant Physiol Biochem 47: 540–549. 19230697 10.1016/j.plaphy.2009.01.004

[10] Sieber P, Schorderet M, Ryser U, Buchala A, Kolattukudy P, et al (2000) Transgenic Arabidopsis plants expressing a fungal cutinase show alterations in the structure and properties of the cuticle and postgenital organ fusions. Plant Cell 12: 721–738. 10810146 10.1105/tpc.12.5.721 PMC139923

[11] Chen X, Goodwin SM, Boroff VL, Liu X, Jenks MA (2003) Cloning and characterization of the *WAX2* gene of Arabidopsis involved in cuticle membrane and wax production. Plant Cell 15: 1170–1185. 12724542 10.1105/tpc.010926 PMC153724

[12] Panikashvili D, Savaldi-Goldstein S, Mandel T, Yifhar T, Franke RB, et al (2007) The Arabidopsis *DESPERADO/AtWBC11* transporter is required for cutin and wax secretion. Plant Physiol 145: 1345–1360. 17951461 10.1104/pp.107.105676 PMC2151707

[13] Bach L, Michaelson LV, Haslam R, Bellec Y, Gissot L, et al (2008) The very-long-chain hydroxy fatty acyl-CoA dehydratase PASTICCINO2 is essential and limiting for plant development. Proc Natl Acad Sci U S A 105: 14727–14731. 18799749 10.1073/pnas.0805089105 PMC2567193

[14] Faure JD, Vittorioso P, Santoni V, Fraisier V, Prinsen E, et al (1998) The *PASTICCINO* genes of *Arabidopsis thaliana* are involved in the control of cell division and differentiation. Development 125: 909–918. 9449673 10.1242/dev.125.5.909

[15] Bellec Y, Harrar Y, Butaeye C, Darnet S, Bellini C, et al (2002) *Pasticcino2* is a protein tyrosine phosphatase-like involved in cell proliferation and differentiation in *Arabidopsis* . Plant J 32: 713–722. 12472687 10.1046/j.1365-313x.2002.01456.x

[16] Haberer G, Erschadi S, Torres-Ruiz RA (2002) The *Arabidopsis* gene *PEPINO/PASTICCINO2* is required for proliferation control of meristematic and non-meristematic cells and encodes a putative anti-phosphatase. Dev Genes Evol 212: 542–550. 12459923 10.1007/s00427-002-0273-9

[17] Harrar Y, Bellec Y, Bellini C, Faure JD (2003) Hormonal control of cell proliferation requires *PASTICCINO* genes. Plant Physiol 132: 1217–1227. 12857804 10.1104/pp.102.019026 PMC167062

[18] Sessions A, Weigel D, Yanofsky MF (1999) The *Arabidopsis thaliana MERISTEM LAYER 1* promoter specifies epidermal expression in meristems and young primordia. Plant J 20: 259–263. 10571886 10.1046/j.1365-313x.1999.00594.x

[19] Baima S, Nobili F, Sessa G, Lucchetti S, Ruberti I, et al (1995) The expression of the *Athb-8* homeobox gene is restricted to provascular cells in *Arabidopsis thaliana* . Development 121: 4171–4182. 8575317 10.1242/dev.121.12.4171

[20] Trenkamp S, Martin W, Tietjen K (2004) Specific and differential inhibition of very-long-chain fatty acid elongases from *Arabidopsis thaliana* by different herbicides. Proc Natl Acad Sci U S A 101: 11903–11908. 15277688 10.1073/pnas.0404600101 PMC511072

[21] Nobusawa T, Umeda M (2012) Very-long-chain fatty acids have an essential role in plastid division by controlling Z-ring formation in *Arabidopsis thaliana* . Genes Cells 17: 709–719. 22734690 10.1111/j.1365-2443.2012.01619.x

[22] Adachi S, Uchimiya H, Umeda M (2006) Expression of B2-type cyclin-dependent kinase is controlled by protein degradation in *Arabidopsis thaliana* . Plant Cell Physiol 47: 1683–1686. 17099223 10.1093/pcp/pcl034

[23] Abe M, Takahashi T, Komeda Y (2001) Identification of a *cis*-regulatory element for L1 layer-specific gene expression, which is targeted by an L1-specific homeodomain protein. Plant J 26: 487–494. 11439135 10.1046/j.1365-313x.2001.01047.x

[24] To JPC, Haberer G, Ferreira FJ, Deruère J, Mason MG, et al (2004) Type-A Arabidopsis response regulators are partially redundant negative regulators of cytokinin signaling. Plant Cell 16: 658–671. 14973166 10.1105/tpc.018978 PMC385279

[25] Miyawaki K, Tarkowski P, Matsumoto-Kitano M, Kato T, Sato S, et al (2006) Roles of *Arabidopsis* ATP/ADP isopentenyltransferases and tRNA isopentenyltransferases in cytokinin biosynthesis. Proc Natl Acad Sci U S A 103: 16598–16603. 17062755 10.1073/pnas.0603522103 PMC1637627

[26] Baud S, Bellec Y, Miquel M, Bellini C, Caboche M, et al (2004) *gurke* and *pasticcino3* mutants affected in embryo development are impaired in acetyl-CoA carboxylase. EMBO Rep 5: 515–520. 15088065 10.1038/sj.embor.7400124 PMC1299045

[27] Smyczynski C, Roudier F, Gissot L, Vaillant E, Grandjean O, et al (2006) The C Terminus of the Immunophilin PASTICCINO1 Is Required for Plant Development and for Interaction with a NAC-like Transcription Factor. J Biol Chem 281: 25475–25484. 16803883 10.1074/jbc.M601815200

[28] Roudier F, Gissot L, Beaudoin F, Haslam R, Michaelson L, et al (2010) Very-long-chain fatty acids are involved in polar auxin transport and developmental patterning in *Arabidopsis* . Plant Cell 22: 364–375. 20145257 10.1105/tpc.109.071209 PMC2845409

[29] Lü S, Zhao H, Parsons EP, Xu C, Kosma DK, et al (2011) The *glossyhead1* allele of *ACC1* reveals a principal role for multidomain acetyl-coenzyme A carboxylase in the biosynthesis of cuticular waxes by Arabidopsis. Plant Physiol 157: 1079–1092. 21949210 10.1104/pp.111.185132 PMC3252135

[30] Lolle SJ, Berlyn GP, Engstrom EM, Krolikowski KA, Reiter WD, Pruiti RE (1997) Developmental regulation of cell interactions in the *Arabidopsis fiddlehead-1* mutant: a role for the epidermal cell wall and cuticle. Dev Biol 189: 311–321. 9299123 10.1006/dbio.1997.8671

[31] Mou Z, He Y, Dai Y, Liu X, Li J (2000) Deficiency in fatty acid synthase leads to premature cell death and dramatic alterations in plant morphology. Plant Cell 12: 405–418. 10715326 10.1105/tpc.12.3.405 PMC139840

[32] Rowland O, Zheng H, Hepworth SR, Lam P, Jetter R, Kunst L (2006) *CER4* encodes an alcohol-forming fatty acyl-coenzyme A reductase involved in cuticular wax production in Arabidopsis. Plant Physiol 142: 866–877. 16980563 10.1104/pp.106.086785 PMC1630741

[33] Greer S, Wen M, Bird D, Wu X, Samuels L, et al (2007) The cytochrome P450 enzyme CYP96A15 is the midchain alkane hydroxylase responsible for formation of secondary alcohols and ketones in stem cuticular wax of Arabidopsis. Plant Physiol 145: 653–667. 17905869 10.1104/pp.107.107300 PMC2048791

[34] Miyawaki K, Matsumoto-Kitano M, Kakimoto T (2004) Expression of cytokinin biosynthetic isopentenyltransferase genes in *Arabidopsis*: tissue specificity and regulation by auxin, cytokinin, and nitrate. Plant J 37: 128–138. 14675438 10.1046/j.1365-313x.2003.01945.x

[35] Takei K, Yamaya T, Sakakibara H (2004) *Arabidopsis CYP735A1* and *CYP735A2* encode cytokinin hydroxylases that catalyze the biosynthesis of *trans*-Zeatin. J Biol Chem 279: 41866–71872. 15280363 10.1074/jbc.M406337200

[36] Culligan KM, Robertson CE, Foreman J, Doerner P, Britt AB (2006) ATR and ATM play both distinct and additive roles in response to ionizing radiation. Plant J 48: 947–961. 17227549 10.1111/j.1365-313X.2006.02931.x

[37] Werner T, Motyka V, Laucou V, Smets R, Van Onckelen H, et al (2003) Cytokinin-deficient transgenic Arabidopsis plants show multiple developmental alterations indicating opposite functions of cytokinins in the regulation of shoot and root meristem activity. Plant Cell 15: 2532–2550. 14555694 10.1105/tpc.014928 PMC280559

[38] Serralbo O, Pérez-Pérez JM, Heidstra R, Scheres B (2006) Non-cell-autonomous rescue of anaphase-promoting complex function revealed by mosaic analysis of *HOBBIT*, an *Arabidopsis CDC27* homolog. Proc Natl Acad Sci U S A 103: 13250–13255. 16938844 10.1073/pnas.0602410103 PMC1559785

[39] Da Costa M, Bach L, Landrieu I, Bellec Y, Catrice O, et al (2006) *Arabidopsis* PASTICCINO2 is an antiphosphatase involved in regulation of cyclin-dependent kinase A. Plant Cell 18: 1426–1437. 16698944 10.1105/tpc.105.040485 PMC1475488

[40] Dissmeyer N, Weimer AK, Pusch S, De Schutter K, Kamei CLA, et al (2009) Control of cell proliferation, organ growth, and DNA damage response operate independently of dephosphorylation of the *Arabidopsis* Cdk1 homolog CDKA;1. Plant Cell 21: 3641–3654. 19948791 10.1105/tpc.109.070417 PMC2798325

[41] Boruc J, Mylle E, Duda M, De Clercq R, Rombauts S, et al (2010) Systematic localization of the Arabidopsis core cell cycle proteins reveals novel cell division complexes. Plant Physiol 152: 553–565. 20018602 10.1104/pp.109.148643 PMC2815867

[42] Galichet A, Hoyerová K, Kamínek M, Gruissem W (2008) Farnesylation directs AtIPT3 subcellular localization and modulates cytokinin biosynthesis in Arabidopsis. Plant Physiol 146: 1155–1164. 18184738 10.1104/pp.107.107425 PMC2259095

[43] Ito Y, Kimura F, Hirakata K, Tsuda K, Takasugi T, et al (2011) Fatty acid elongase is required for shoot development in rice. Plant J 66: 680–688. 21309865 10.1111/j.1365-313X.2011.04530.x

[44] Yanai O, Shani E, Dolezal K, Tarkowski P, Sablowski R, et al (2005) *Arabidopsis* KNOXI proteins activate cytokinin biosynthesis. Curr Biol 15: 1566–1571. 16139212 10.1016/j.cub.2005.07.060

[45] Rupp HM, Frank M, Werner T, Strnad M, Schmülling T (1999) Increased steady state mRNA levels of the *STM* and *KNAT1* homeobox genes in cytokinin overproducing *Arabidopsis thaliana* indicate a role for cytokinins in the shoot apical meristem. Plant J 18: 557–563. 10417706 10.1046/j.1365-313x.1999.00472.x

[46] Chen M, Han G, Dietrich CR, Dunn TM, Cahoon EB (2006) The essential nature of sphingolipids in plants as revealed by the functional identification and characterization of the *Arabidopsis* LCB1 subunit of serine palmitoyltransferase. Plant Cell 18: 3576–3593. 17194770 10.1105/tpc.105.040774 PMC1785403

[47] Dietrich CR, Han G, Chen M, Berg RH, Dunn TM, et al (2008) Loss-of-function mutations and inducible RNAi suppression of Arabidopsis *LCB2* genes reveal the critical role of sphingolipids in gametophytic and sporophytic cell viability. Plant J 54: 284–298. 18208516 10.1111/j.1365-313X.2008.03420.x

[48] Black PN, Faergeman NJ, DiRusso CC (2000) Long-chain acyl-CoA-dependent regulation of gene expression in bacteria, yeast and mammals. J Nutr 130: 305S–309S. 10721893 10.1093/jn/130.2.305S

[49] Savchenko T, Walley JW, Chehab EW, Xiao Y, Kaspi R, et al (2010) Arachidonic acid: An evolutionarily conserved signaling molecule modulates plant stress signaling networks. Plant Cell 22: 3193–3205. 20935246 10.1105/tpc.110.073858 PMC2990140

[50] Joubès J, Raffaele S, Bourdenx B, Garcia C, Laroche-Traineau J, et al (2008) The VLCFA elongase gene family in *Arabidopsis thaliana*: phylogenetic analysis, 3D modelling and expression profiling. Plant Mol Biol 67: 547–566. 18465198 10.1007/s11103-008-9339-z

[51] Fiebig A, Mayfield JA, Miley NL, Chau S, Fischer RL, et al (2000) Alterations in *CER6*, a gene identical to *CUT1*, differentially affect long-chain lipid content on the surface of pollen and stems. Plant Cell 12: 2001–2008. 11041893 10.1105/tpc.12.10.2001 PMC149136

[52] Hooker TS, Millar AA, Kunst L (2002) Significance of the expression of the CER6 condensing enzyme for cuticular wax production in Arabidopsis. Plant Physiol 129: 1568–1580. 12177469 10.1104/pp.003707 PMC166744

[53] Yephremov A, Wisman E, Huijser P, Huijser C, Wellesen K, et al (1999) Characterization of the *FIDDLEHEAD* gene of Arabidopsis reveals a link between adhesion response and cell differentiation in the epidermis. Plant Cell 11: 2187–2201. 10559443 10.1105/tpc.11.11.2187 PMC144117

[54] Lee SB, Jung SJ, Go YS, Kim HU, Kim JK, et al (2009) Two Arabidopsis 3-ketoacyl CoA synthase genes, *KCS20* and *KCS2/DAISY*, are functionally redundant in cuticular wax and root suberin biosynthesis, but differentially controlled by osmotic stress. Plant J 60: 462–475. 19619160 10.1111/j.1365-313X.2009.03973.x

[55] Bemis SM, Torii KU (2007) Autonomy of cell proliferation and developmental programs during *Arabidopsis* aboveground organ morphogenesis. Dev Biol 304: 367–381. 17258192 10.1016/j.ydbio.2006.12.049

[56] Raffaele S, Vailleau F, Léger A, Joubès J, Miersch O, et al (2008) A MYB transcription factor regulates very-long-chain fatty acid biosynthesis for activation of the hypersensitive cell death response in *Arabidopsis* . Plant Cell 20: 752–767. 18326828 10.1105/tpc.107.054858 PMC2329921

[57] Li L, Yu X, Thompson A, Guo M, Yoshida S, et al (2009) Arabidopsis MYB30 is a direct target of BES1 and cooperates with BES1 to regulate brassinosteroid-induced gene expression. Plant J 58: 275–286. 19170933 10.1111/j.1365-313X.2008.03778.x PMC2814797

[58] Nakagawa T, Kurose T, Hino T, Tanaka K, Kawamukai M, et al (2007) Development of series of Gateway Binary Vectors, pGWBs, for realizing efficient construction of fusion genes for plant transformation. J Biosci Bioeng 104: 34–41. 17697981 10.1263/jbb.104.34

[59] Wesley SV, Helliwell CA, Smith NA, Wang MB, Rouse DT, et al (2001) Construct design for efficient, effective and high-throughput gene silencing in plants. Plant J 27: 581–590. 11576441 10.1046/j.1365-313x.2001.01105.x

[60] Gleave AP (1992) A versatile binary vector system with a T-DNA organisational structure conducive to efficient integration of cloned DNA into the plant genome. Plant Mol Biol 20: 1203–1207. 1463857 10.1007/BF00028910

[61] Nakagawa T, Nakamura S, Tanaka K, Kawamukai M, Suzuki T, et al (2008) Development of R4 gateway binary vectors (R4pGWB) enabling high-throughput promoter swapping for plant research. Biosci Biotechnol Biochem 72: 624–629. 18256458 10.1271/bbb.70678

[62] Adachi S, Nobusawa T, Umeda M (2009) Quantitative and cell type-specific transcriptional regulation of A-type cyclin-dependent kinase in *Arabidopsis thaliana* . Dev Biol 329: 306–314. 19285489 10.1016/j.ydbio.2009.03.002

[63] Kojima M, Kamada-Nobusada T, Komatsu H, Takei K, Kuroha T, et al (2009) Highly-sensitive and high-throughput analysis of plant hormones using MS-probe modification and liquid chromatography-tandem mass spectrometry: an application for hormone profiling in *Oryza sativa* . Plant Cell Physiol 50: 1201–1214. 19369275 10.1093/pcp/pcp057 PMC2709547

