# Supplementary figures and images for: Synthesis of Very-Long-Chain Fatty Acids in the Epidermis Controls Plant Organ Growth by Restricting Cell Proliferation
Source: PLoS Biol. 2013 Apr 9;11(4):e1001531. doi: 10.1371/journal.pbio.1001531 (PMC3621670; doi:10.1371/journal.pbio.1001531)

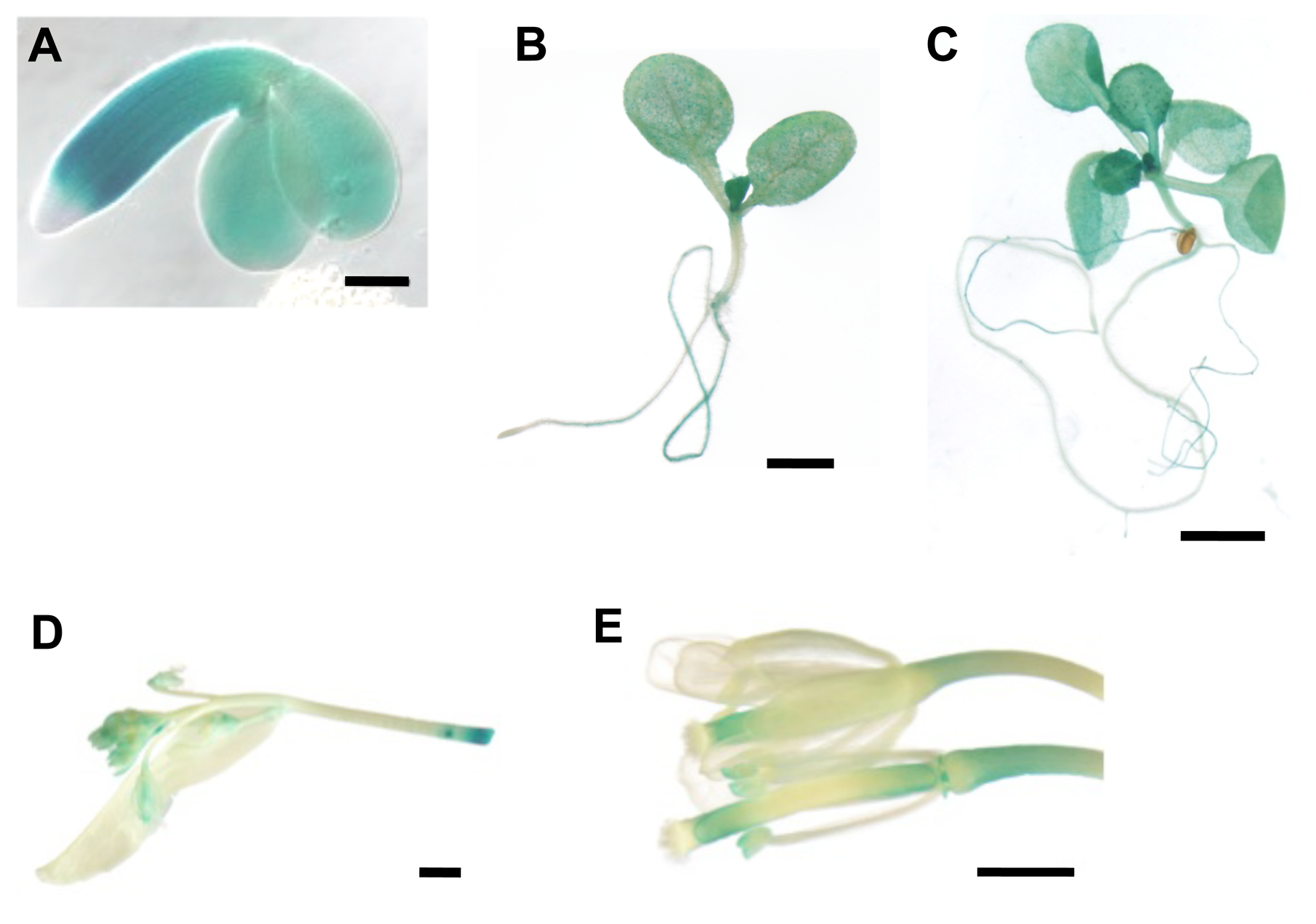

Supplement: Figure S1 — Expression pattern of PAS2. GUS staining of transgenic plants carrying ProPAS2:GUS. Mature embryo (A), 5-d-old seedling (B), 8-d-old seedling (C), inflorescence (D), and flowers and anthers (E). Bars, 100 µm (A), 1 mm (B, E), and 2 mm (C, D). (TIF) [file pbio.1001531.s001.tif]

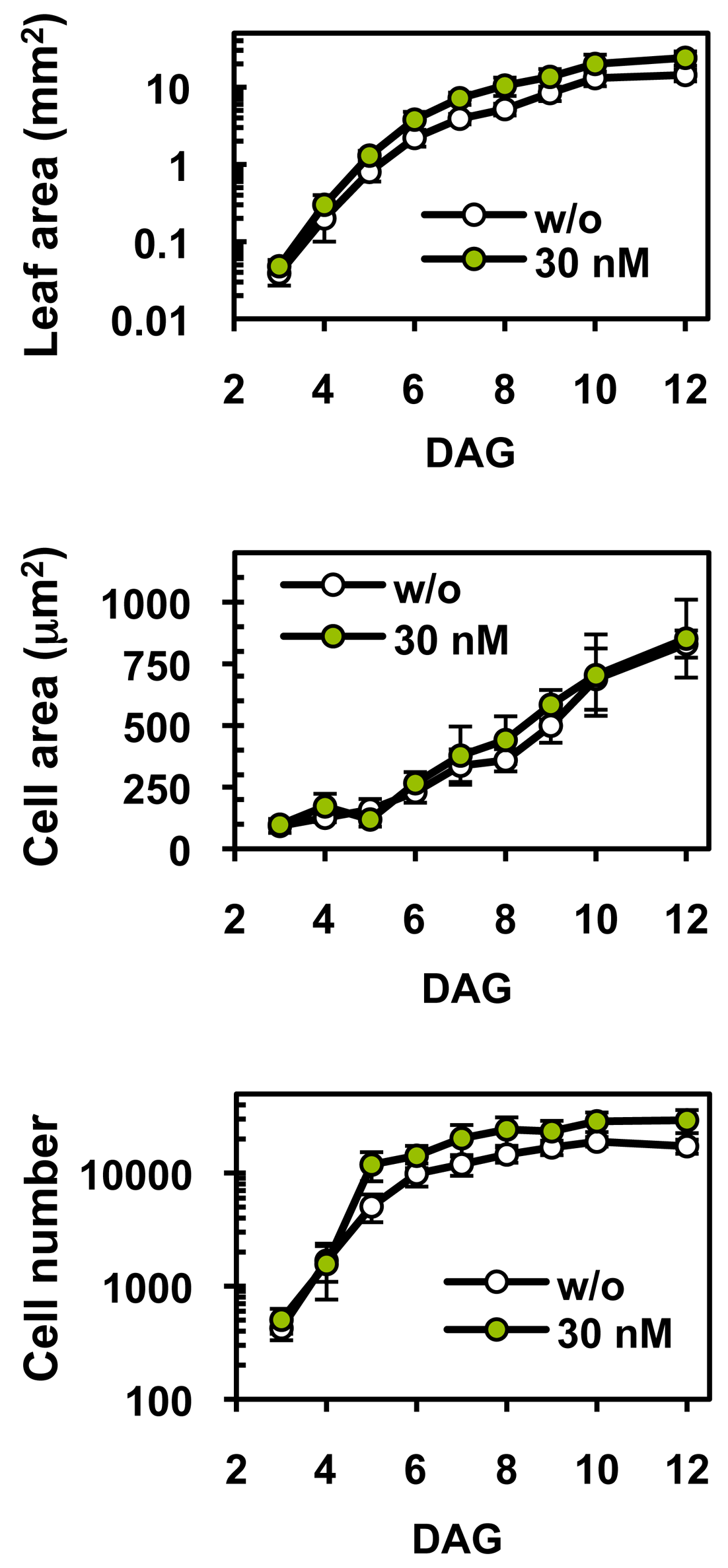

Supplement: Figure S2 — Kinematic analysis of leaf growth. First leaves of wild-type seedlings grown in the absence (w/o) or presence of 30 nM cafenstrole were measured for leaf blade area, cell area, and cell number per leaf. Data are presented as mean ± SD (n≥10). DAG, days after germination. (TIF) [file pbio.1001531.s002.tif]

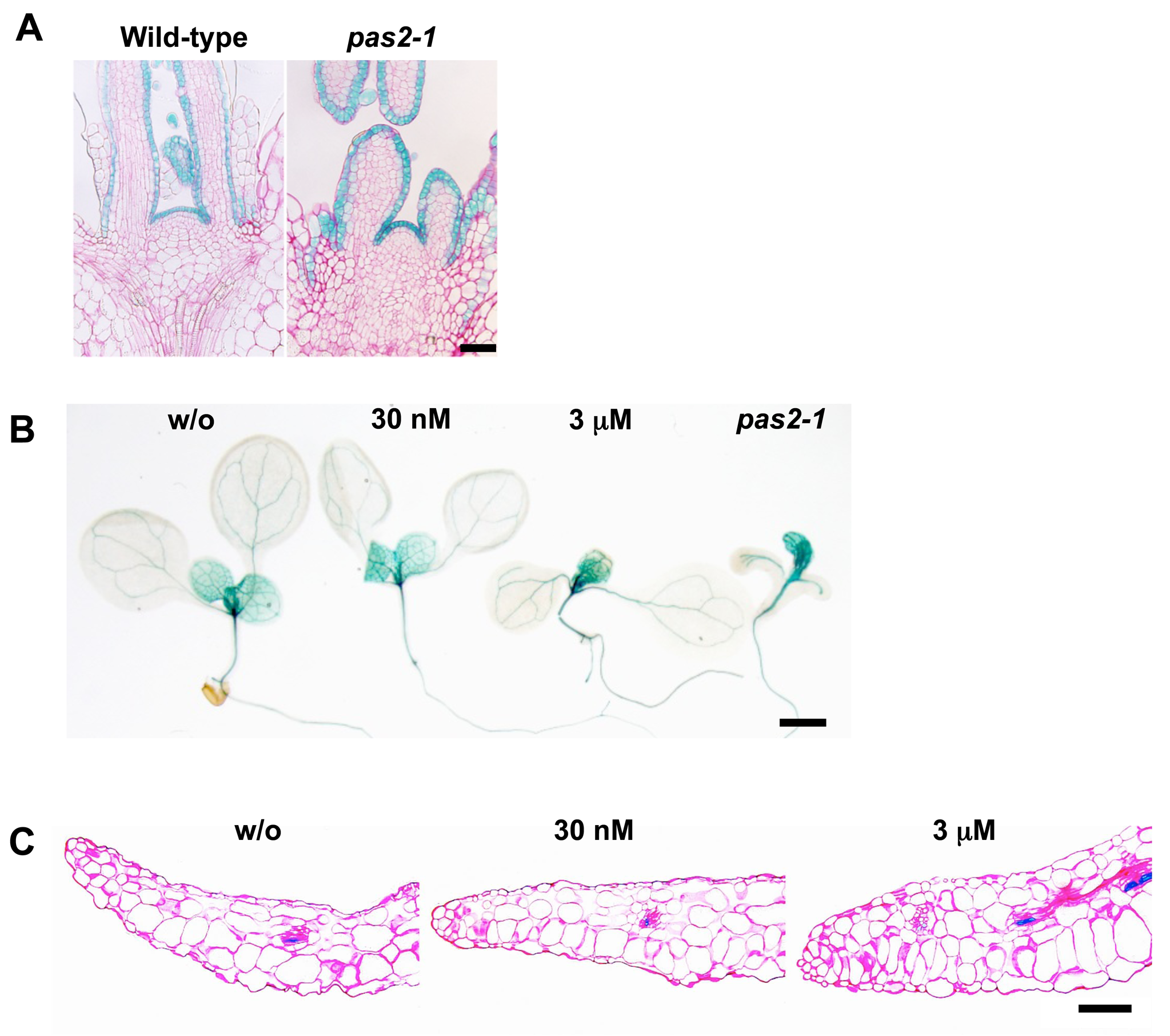

Supplement: Figure S3 — Reduced VLCFA synthesis does not affect the expression patterns of PDF1 and ATHB8. (A) Expression pattern of ProPDF1:GUS in wild-type and pas2-1. Transverse sections of shoot apices of 5-d-old seedlings. (B, C) Expression pattern of ProATHB8:GUS. 5-d-old wild-type seedlings grown in the absence (w/o) or presence of cafenstrole (30 nM or 3 µM) (B) and cross sections of cotyledons (C). pas2-1 grown in the absence of cafenstrole is shown for comparison. Bars, 50 µm (A), 1 mm (B), and 100 µm (C). (TIF) [file pbio.1001531.s003.tif]

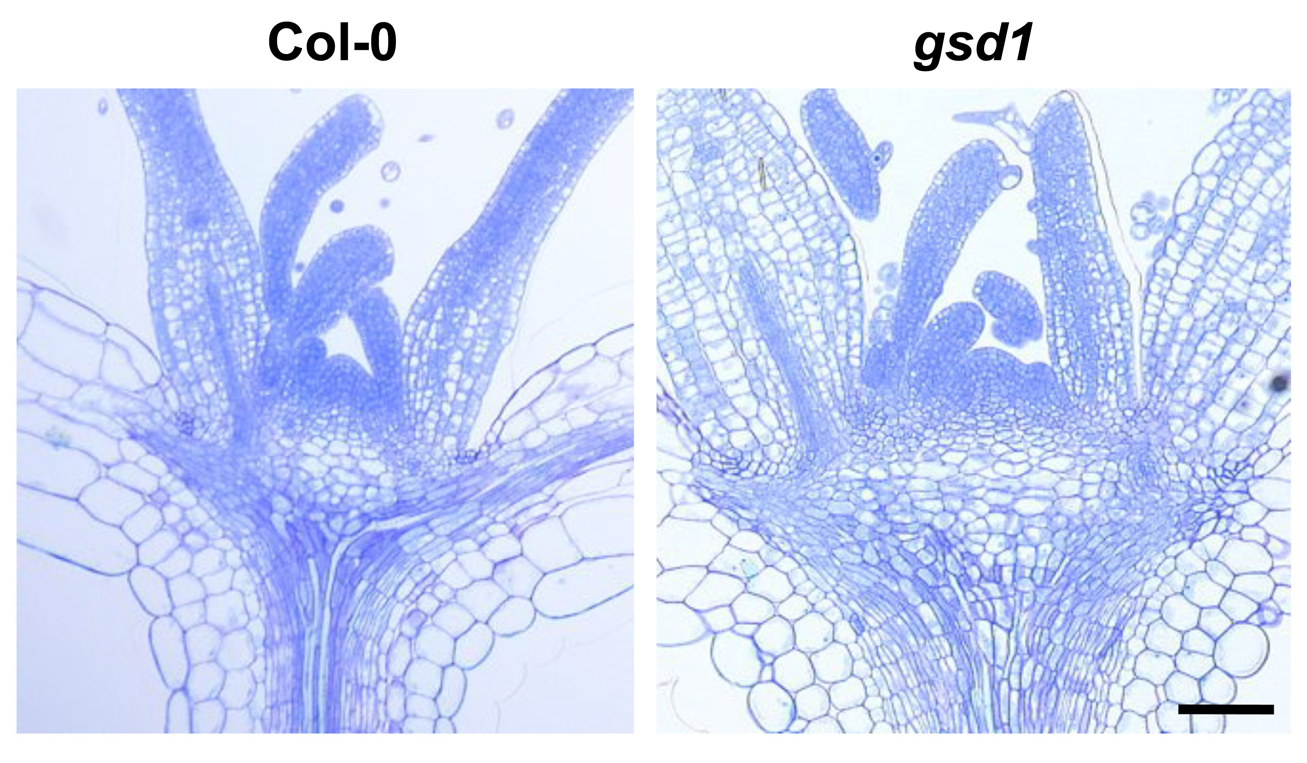

Supplement: Figure S4 — Enhanced cell proliferation in the gsd1 mutant. Transverse sections of shoot apices of 7-d-old Col-0 and gsd1 seedlings. Bar, 100 µm. (TIF) [file pbio.1001531.s004.tif]

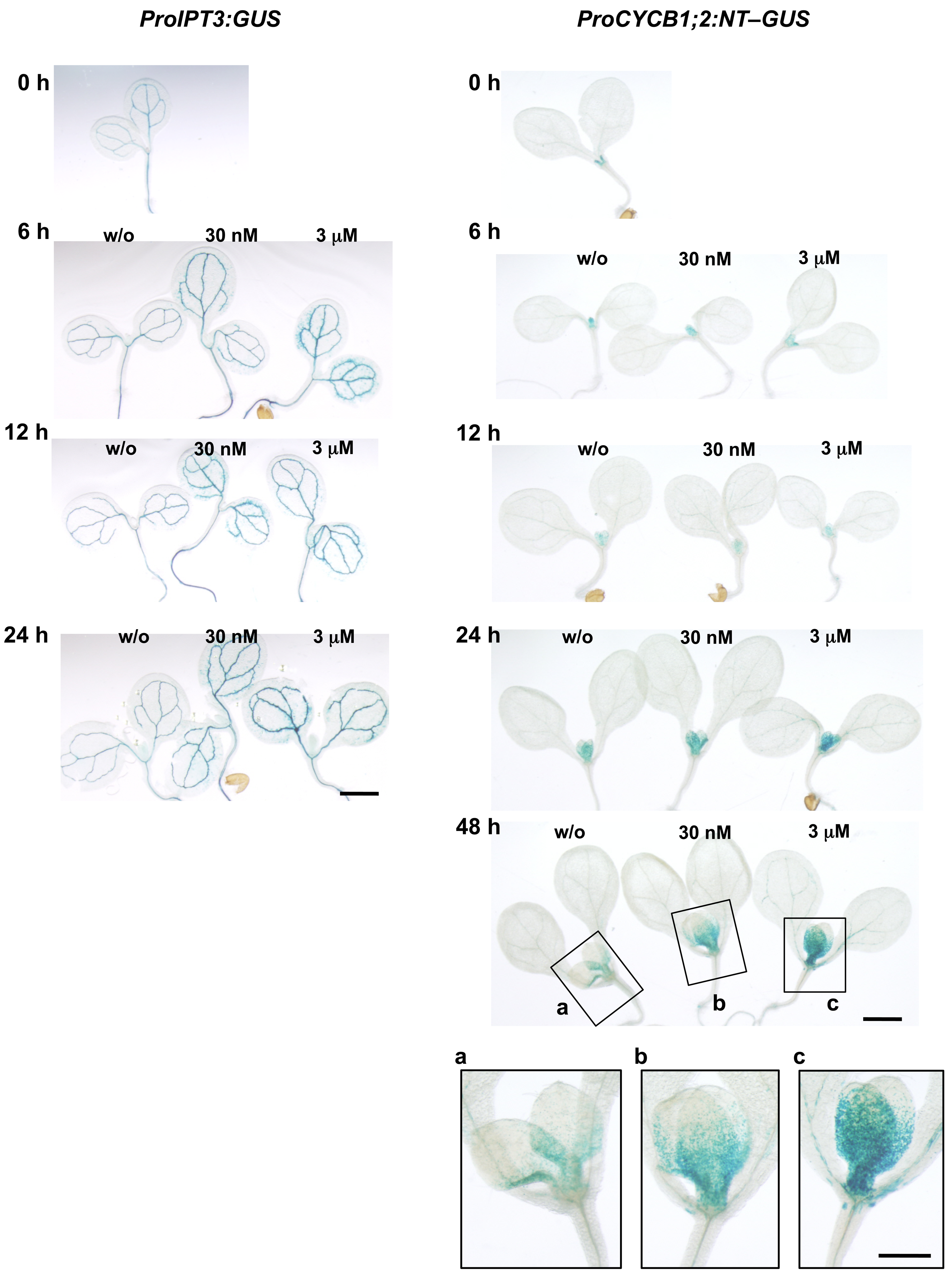

Supplement: Figure S5 — IPT3 and CYCB1;2 expression after cafenstrole treatment. 3-d-old seedlings carrying ProIPT3:GUS or ProCYCB1;2:NT–GUS were transferred onto a medium without cafenstrole (w/o), or containing 30 nM or 3 µM cafenstrole, and GUS expression was observed at the indicated time points thereafter. Enlarged images of ProCYCB1;2:NT–GUS seedlings after 48 h are shown below (a–c). Bars, 1 mm and 500 µm (a–c). (TIF) [file pbio.1001531.s005.tif]

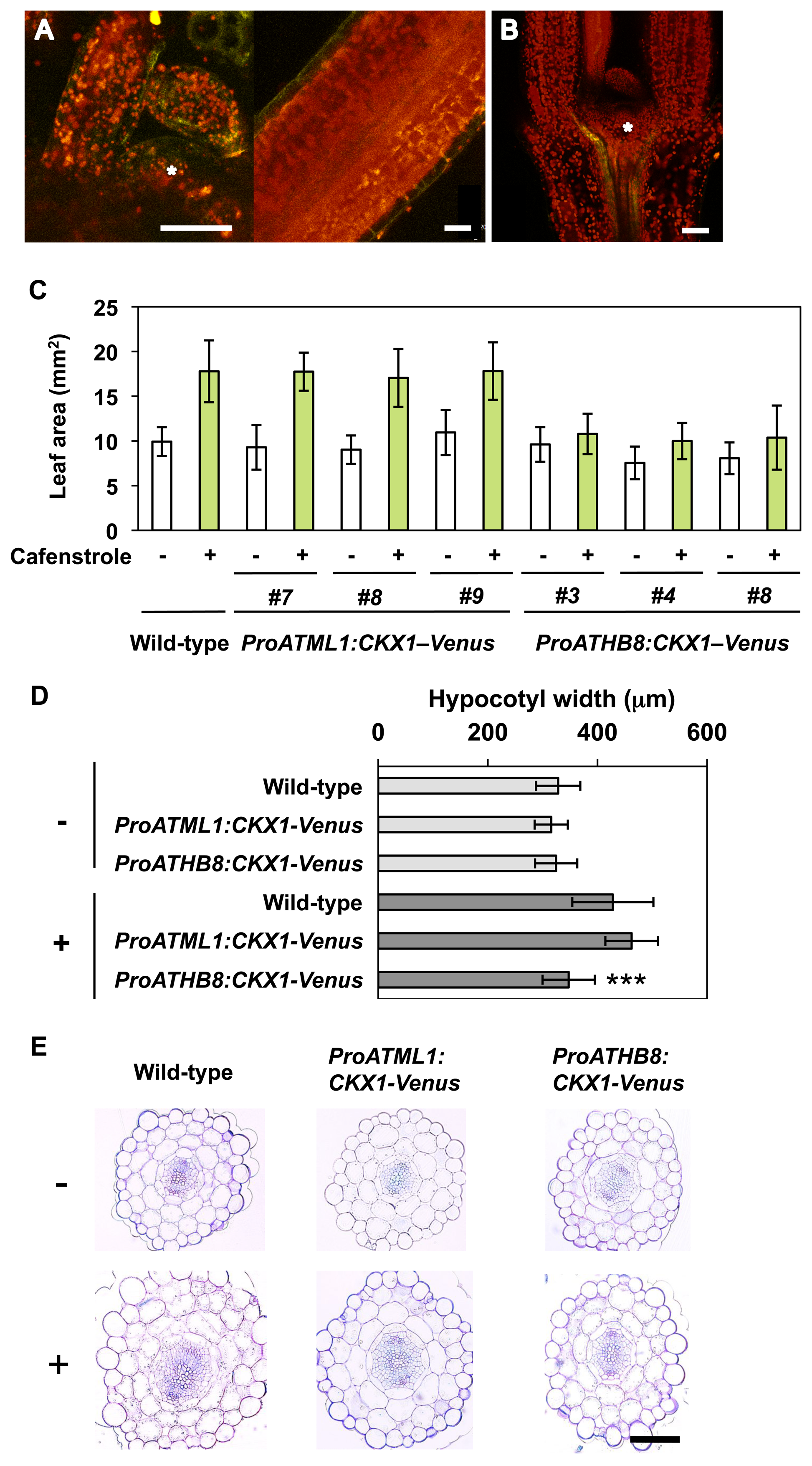

Supplement: Figure S6 — Vasculature-specific expression of CKX1 suppresses the increase of hypocotyl width caused by cafenstrole treatment. (A, B) Expression patterns of CKX1–Venus controlled by the ATML1 (A) and ATHB8 (B) promoters. Transverse sections of shoot apices (left image of A, B) and cross section of a first leaf (right image of A). Venus fluorescence was merged with autofluorescence. Asterisks indicate the SAM. (C) First leaves of 10-d-old seedlings of wild-type, ProATML1:CKX1–Venus and ProATHB8:CKX1–Venus grown in the absence (white bars) or presence (green bars) of 30 nM cafenstrole were measured for leaf blade area. For each promoter construct, three independent lines, which are different from those shown in Figure 9, were used for measurement. Data are presented as mean ± SD (n≥20). (D) Measurement of hypocotyl width. Hypocotyls of 8-d-old seedlings grown in the absence (−) or presence (+) of 30 nM cafenstrole were measured. Data are presented as mean ± SD (n≥20). Significant differences between wild-type and CKX1–Venus transgenic seedlings were determined by Student's t-test: ***, p<0.001; the other differences are not significant (p>0.05). (E) Cross sections of 5-d-old hypocotyls grown in the absence (−) or presence (+) of 30 nM cafenstrole. Bars, 50 µm (A), 20 µm (B), and 100 µm (E). (TIF) [file pbio.1001531.s006.tif]
