# Supplementary material for: Synthesis of Very-Long-Chain Fatty Acids in the Epidermis Controls Plant Organ Growth by Restricting Cell Proliferation
Source: PLoS Biol. 2013 Apr 9;11(4):e1001531. doi: 10.1371/journal.pbio.1001531 (PMC3621670; doi:10.1371/journal.pbio.1001531)
Supplement: Table S1 — Expression levels of cytokinin biosynthesis genes in the pas2-1 mutant. Average values of biological duplicates in microarray analysis are shown as relative values, with those for wild-type set to 1. (DOCX) [file pbio.1001531.s007.docx]

| **Table S1.** Expression levels of cytokinin biosynthesis genes in the *pas2-1* mutant. | |
| --- | --- |
| Cytokinin  biosynthesis genes | Fold change  *(pas2-1*/wild-type) |
| *IPT1* | 0.9 |
| *IPT2* | 1.0 |
| *IPT3* | 3.9 |
| *IPT4* | 1.6 |
| *IPT5* | 0.8 |
| *IPT6* | 0.9 |
| *IPT7* | 1.5 |
| *IPT8* | 0.6 |
| *IPT9* | 1.0 |
| *CYP735A1* | 0.8 |
| *CYP735A2* | 6.6 |
| *URH1* | 1.5 |
|  |  |
| Average values of biological duplicates in microarray analysis are shown as relative values, with those for wild-type set to 1. | |
