# Supplementary material for: Synthesis of Very-Long-Chain Fatty Acids in the Epidermis Controls Plant Organ Growth by Restricting Cell Proliferation
Source: PLoS Biol. 2013 Apr 9;11(4):e1001531. doi: 10.1371/journal.pbio.1001531 (PMC3621670; doi:10.1371/journal.pbio.1001531)
Supplement: Table S2 — Cytokinin contents after cafenstrole treatment. 3-d-old wild-type seedlings were transferred onto a medium without cafenstrole (w/o), or containing 30 nM or 3 µM cafenstrole, and measured for cytokinin contents after indicated time points. Data are presented as mean (pmol/g fresh weight) ± SD (n = 3). (DOCX) [file pbio.1001531.s008.docx]

| **Table S2.** Cytokinin content after cafenstrole treatment. | | | | | | | | | |  |  |  |  |  |  |
| --- | --- | --- | --- | --- | --- | --- | --- | --- | --- | --- | --- | --- | --- | --- | --- |
| **iPRPs** |  |  |  |  |  |  |  |  |  |  |  |  |  |  |  |
|  | 0 h | | |  | 6 h | | |  | 12 h | | |  | 24 h | | |
| w/o | 30.70 | ± | 2.23 |  | 36.43 | ± | 2.78 |  | 38.78 | ± | 10.82 |  | 33.07 | ± | 2.89 |
| 30 nM |  |  |  |  | 47.64 | ± | 3.88 |  | 41.64 | ± | 7.36 |  | 46.46 | ± | 11.17 |
| 3 μM |  |  |  |  | 48.05 | ± | 13.40 |  | 77.63 | ± | 15.76 |  | 129.41 | ± | 33.31 |
| **iPR** |  |  |  |  |  |  |  |  |  |  |  |  |  |  |  |
|  | 0 h | | |  | 6 h | | |  | 12 h | | |  | 24 h | | |
| w/o | 0.20 | ± | 0.07 |  | 0.14 | ± | 0.03 |  | 0.15 | ± | 0.05 |  | 0.15 | ± | 0.05 |
| 30 nM |  |  |  |  | 0.20 | ± | 0.06 |  | 0.17 | ± | 0.03 |  | 0.23 | ± | 0.08 |
| 3 μM |  |  |  |  | 0.21 | ± | 0.02 |  | 0.30 | ± | 0.06 |  | 0.43 | ± | 0.05 |
| **iP** |  |  |  |  |  |  |  |  |  |  |  |  |  |  |  |
|  | 0 h | | |  | 6 h | | |  | 12 h | | |  | 24 h | | |
| w/o | 0.60 | ± | 0.15 |  | 0.49 | ± | 0.05 |  | 0.52 | ± | 0.09 |  | 0.48 | ± | 0.01 |
| 30 nM |  |  |  |  | 0.56 | ± | 0.05 |  | 0.52 | ± | 0.04 |  | 0.54 | ± | 0.09 |
| 3 μM |  |  |  |  | 0.54 | ± | 0.09 |  | 0.67 | ± | 0.05 |  | 0.74 | ± | 0.07 |
| **tZRPs** |  |  |  |  |  |  |  |  |  |  |  |  |  |  |  |
|  | 0 h | | |  | 6 h | | |  | 12 h | | |  | 24 h | | |
| w/o | 6.74 | ± | 1.22 |  | 16.45 | ± | 3.65 |  | 21.12 | ± | 6.47 |  | 14.70 | ± | 2.14 |
| 30 nM |  |  |  |  | 20.57 | ± | 3.37 |  | 23.26 | ± | 4.40 |  | 22.15 | ± | 0.51 |
| 3 μM |  |  |  |  | 18.48 | ± | 6.73 |  | 30.96 | ± | 5.31 |  | 57.45 | ± | 10.35 |
| **tZR** |  |  |  |  |  |  |  |  |  |  |  |  |  |  |  |
|  | 0 h | | |  | 6 h | | |  | 12 h | | |  | 24 h | | |
| w/o | 1.06 | ± | 0.29 |  | 1.29 | ± | 0.20 |  | 1.43 | ± | 0.43 |  | 1.08 | ± | 0.25 |
| 30 nM |  |  |  |  | 1.57 | ± | 0.11 |  | 1.69 | ± | 0.19 |  | 2.01 | ± | 0.27 |
| 3 μM |  |  |  |  | 1.71 | ± | 0.51 |  | 3.45 | ± | 0.86 |  | 6.35 | ± | 1.12 |
| **tZ** |  |  |  |  |  |  |  |  |  |  |  |  |  |  |  |
|  | 0 h | | |  | 6 h | | |  | 12 h | | |  | 24 h | | |
| w/o | 0.60 | ± | 0.21 |  | 0.72 | ± | 0.07 |  | 0.85 | ± | 0.18 |  | 0.67 | ± | 0.06 |
| 30 nM |  |  |  |  | 0.85 | ± | 0.06 |  | 0.93 | ± | 0.21 |  | 0.86 | ± | 0.02 |
| 3 μM |  |  |  |  | 0.75 | ± | 0.19 |  | 0.96 | ± | 0.14 |  | 1.25 | ± | 0.08 |

Three-day-old wild-type seedlings were transferred onto a medium without cafenstrole (w/o), or containing 30 nM or 3 µM cafenstrole, and measured for cytokinin content after the indicated time points. Data are presented as mean (pmol/g fresh weight) ± s.d. (n = 3).
