# Supplementary material for: Synthesis of Very-Long-Chain Fatty Acids in the Epidermis Controls Plant Organ Growth by Restricting Cell Proliferation
Source: PLoS Biol. 2013 Apr 9;11(4):e1001531. doi: 10.1371/journal.pbio.1001531 (PMC3621670; doi:10.1371/journal.pbio.1001531)
Supplement: Table S3 — Primers used for plasmid constructions. CDS (coding sequence) was PCR-amplified from the genomic DNA. (DOCX) [file pbio.1001531.s009.docx]

**Table S3.** Primers used for plasmid constructions.

| DNA fragment  (Construct) | Forward primer (5′–3′) |
| --- | --- |
|  | Reverse primer (5′–3′) |
| *PAS2* promoter  (*ProPAS2:GUS*) | AGTCGACCGGAATATCCACTGTTAGCTTTAGACG |
|  | AGGATCCATGGAAAGTGAATACGCGAGAACGAC |
| *ProPAS2:PAS2*  (*ProPAS2:PAS2–GUS*) | GGGGACAAGTTTGTACAAAAAAGCAGGCTGCCGGAATATCCACTGTTAGCTTTAGACGTTCC |
|  | GGGGACCACTTTGTACAAGAAAGCTGGGTGTTCCCTCTTGGATTTGGAGAGAGCTCTC |
| *ATML1* promoter  (*ProATML1:PAS2* and *ProATML1:PAS2RNAi*) | AGAATTCATTGATTCTGAACTGTACCC |
|  | TGAATTCAACCGGTGGATTCAGGG |
| *PAS2* CDS  (*ProATML1:PAS2–GUS*) | ATGGCGGGCTTTCTCTCCGTT |
|  | TTCCCTCTTGGATTTGGAGAGAGCTC |
| *PAS2* ORF No. 6-641  sense (*PAS2RNAi*) | AGAATTCGGGCTTTCTCTCCGTTGTCC |
|  | AGGTACCGCTCTCTTTCGCTGACCAAGC |
| *PAS2* ORF No. 6-641 antisense (*PAS2RNAi*) | AGGATCCGGGCTTTCTCTCCGTTGTCC |
|  | AAAGCTTGCTCTCTTTCGCTGACCAAGC |
| *Venus* ORF  (*CKX1–Venus*) | AAAGTCGACATGGTGAGCAAGGGCG |
|  | ACTCGAGTTACTTGTACAGCTCGTCCATG |
| *CKX1* CDS  (*CKX1–Venus*) | AGTCGACATGGGATTGACCTCATCCTTAC |
|  | AGTCGACTACAGTTCTAGGTTTCGGCAGTAT |
| *CKX1–Venus*  (*ProATML1:CKX1–Venus* and *ProATHB8:CKX1–Venus*) | GGGGACAAGTTTGTACAAAAAAGCAGGCTGCATGGGATTGACCTCATCCTTAC |
|  | GGGGACCACTTTGTACAAGAAAGCTGGGTCTTACTTGTACAGCTCGTCCATG |
| *ATML1* promoter  (*ProATML1:CKX1–Venus,*  *ProATML1:PAS2–GUS and ProATML1:PAS2RNAi*) | GGGGACAACTTTGTATAGAAAAGTTGATTGATTCTGAACTGTACCC |
|  | GGGGACTGCTTTTTTGTACAAACTTGAACCGGTGGATTCAGGGAG |
| *ATHB8* promoter  (*ProATHB8:CKX1–Venus,*  *ProATHB8:PAS2–GUS and ProATHB8:PAS2RNAi*) | GGGGACAACTTTGTATAGAAAAGTTGCGGATAAACCAATTTTCAAATGATA |
|  | GGGGACTGCTTTTTTGTACAAACTTGCTTTGATCCTCTCCGATCTCTCTAT |
| *PAS2–GUS*  (*ProATML1:PAS2–GUS and ProATHB8:PAS2–GUS*) | GGGGACAAGTTTGTACAAAAAAGCAGGCTGCATGGCGGGCTTTCTCTCCGT |
|  | GGGGACCACTTTGTACAAGAAAGCTGGGTCTCATTGTTTGCCTCCCTGCTGC |
| *PAS2RNAi*  (*ProATML1:PAS2RNAi and ProATHB8:PAS2RNAi*) | GGGGACAAGTTTGTACAAAAAAGCAGGCTGCGGGCTTTCTCTCCGTTGTCC |
|  | GGGGACCACTTTGTACAAGAAAGCTGGGTCGCTCTCTTTCGCTGACCAAGC |

CDS (coding sequence) was PCR-amplified from the genomic DNA. ORF, open reading frame.
